# Licking microstructure behavior classifies a spectrum of emotional states in mice

**DOI:** 10.3389/fnsys.2025.1623084

**Published:** 2025-08-13

**Authors:** Randa Salalha, Micky Holzman, Federica Cruciani, Gil Ben David, Yam Amir, Firas Mawase, Kobi Rosenblum

**Affiliations:** ^1^Sagol Department of Neuroscience, The Integrated Brain and Behavior Center, University of Haifa, Haifa, Israel; ^2^Department of Human Biology, Faculty of Natural Sciences, University of Haifa, Haifa, Israel; ^3^Faculty of Biomedical Engineering, Technion–Israel Institute of Technology, Haifa, Israel; ^4^Center for Gene Manipulation in the Brain, University of Haifa, Haifa, Israel

**Keywords:** taste valence, licking microstructure, ingestive behavior, emotional states, salience

## Abstract

Measuring precise emotional tagging for taste information, with or without the use of words, is challenging. While affective taste valence and salience are core components of emotional experiences, traditional behavioral assays for taste preference, which often rely on cumulative consumption, lack the resolution to distinguish between different affective states, such as innate versus learned aversion, which are known to be mediated by distinct neural circuits. To overcome this limitation, we developed an open-source system for high-resolution microstructural analysis of licking behavior in freely moving mice. Our approach integrates traditional lick burst analysis with a proprietary software pipeline that utilizes interlick interval (ILI) distributions and principal component analysis (PCA) to create a multidimensional behavioral profile of the animal. Using this system, we characterized the licking patterns associated with innate appetitive, aversive, and neutral tastants. While conventional burst analysis failed to differentiate between two palatable stimuli (water and saccharin), our multidimensional approach revealed distinct and quantifiable behavioral signatures for each. Critically, this approach successfully dissociates innate and learned aversive taste valences, a distinction that cannot be achieved using standard metrics. By providing the designs for our custom-built setup and analysis software under an open-source license, this study offers a comprehensive and accessible methodology for examining hedonic responses in future studies. This powerful toolkit enhances our understanding of sensory valence processing and provides a robust platform for future investigations of the neurobiology of ingestive behavior.

## Significance statement

We can ask what our taste is (i.e., do we like, dislike, or anything in between) about something, but an equally important question is how we define and measure the emotional tag for a given taste. Here, we measured taste valence and saliency using microstructural analysis of licking behavior in mice as an important step toward better identification of the cellular and molecular mechanisms underlying the emotional tag associated with taste. Our custom-made setup allowed freely behaving mice to voluntarily consume tastants and record their licking behavior with high temporal resolution. Integrating the temporal characteristics of bursts, frequency distribution of interlick intervals, and multivariate analysis methods allowed us to identify distinct patterns of licking behavior among innate appetitive (saccharin), innate aversive (quinine), and neutral (water) tastants. Furthermore, our analysis revealed differential licking patterns associated with innate and learned aversive taste valences.

## Introduction

The emotional world is complex and comprises various components. Measuring objective and precise behavioral responses to emotional states is a significant challenge, particularly in animal models (Adolphs and Anderson, 2018; Gu et al., 2018). Affective valence is a primary dimension of emotional experience (Itkes et al., 2017) that determines how individuals evaluate their emotional state (Harmon-Jones et al., 2017). Valence represents the hedonic value of a stimulus, which can be pleasurable (positive) or aversive (negative). The assignment of emotional valence to each stimulus is necessary for survival, adaptive reactions, and predicting future states in a changing environment. Positive stimuli are approached again in subsequent experiences, and aversive stimuli are avoided (Bilman et al., 2017; Nicolas et al., 2023; Tye, 2018). However, valence is not limited to the designation of values at opposite ends of the same dimension and can be conceptualized at multiple levels rather than as a single dimension, as suggested by appraisal theories (Shuman et al., 2013).

Innate and acquired valence are closely related to an individual’s personality and performance. Psychiatric disorders involve deficits in emotional valence processing and incorrect assignment of stimuli as positive or negative. Affective disorders, such as depression and schizophrenia, can cause anhedonia (Dwyer, 2012; Pandurangan and Hwang, 2015). Fluid consumption has been widely used to assess affective states in both rodents and humans (Gero, 2020). In addition to regulating nutrient intake, fluid consumption reflects the innate or learned emotional tag associated with a particular taste and post-intestinal experiences, as well as preferences, aversions, and internal states (Dwyer, 2012; Pandurangan and Hwang, 2015). Updating taste valence is a behavior preserved through evolution that is influenced by factors such as stimulus concentration, experience, and internal state (Mi et al., 2021; Tye, 2018). Some taste stimuli are innately aversive or appetitive and require no prior experience to attribute emotional tags. Learned valence can be created through associations between a conditioned stimulus (CS) and a subsequent response, such as conditioned taste aversion (CTA) (Revusky and Garcia, 1970; Rosenblum et al., 1993), a known model for studying associative learning, wherein an initially appetitive tastant is associated with unpleasant gastrointestinal malaise, which causes a valence shift from appetitive to aversive (Lavi et al., 2018). This form of learning is crucial for the survival of animals because it allows them to avoid potentially harmful substances in their environment (Yiannakas and Rosenblum, 2017). Taste memories are often retrieved with other information, resulting in behaviors that reflect taste identity and subjective perception of taste value for various experiences (Yiannakas et al., 2021).

Most taste valence coding studies have used overall intake as the sole variable metric in single- or multiple-choice tests. However, this measure lacks the ability to examine the rich spectrum of emotional tagging that drives consumption behavior (Johnson, 2018). In taste aversion learning, the CTA lowers the hedonic value of taste (palatability), which is measured by the overall intake. A critical aspect of this paradigm is its ability to change the palatability of a tastant without altering its perceived sensory qualities such as identity or intensity (Fonseca et al., 2020; Wang et al., 2018). For instance, CTA can invert the hedonic valence of sucrose from positive to negative, while leaving the perception of its sweet intensity intact (Fonseca et al., 2020). This demonstrates that CTA can be used to specifically manipulate the valence attributes of a tastant, allowing for an experimental dissociation of its hedonic value from its perceived intensity (Fonseca et al., 2020; Small et al., 2003). Animals also consume less quinine, which is an innately aversive tastant (Berridge and Kringelbach, 2015; Lin et al., 2014).

Rodents consume fluids in a stereotypical manner, consisting of runs of rapid rhythmic licks (referred to here as “bursts”) separated by pauses (Davis and Smith, 1992). This process differs from receiving taste stimuli through intraoral cannulas (Levitan et al., 2019; Stapleton et al., 2007) or anesthetized preparations (Nishijo et al., 1998). Microstructural analysis of licking during voluntary drinking is a precise and valid tool for assessing hedonic taste reactions in rats. This approach allows the identification of physiological and psychological parameters and subjective experiences underlying taste behavior in rats and humans (Gero, 2020; Naneix et al., 2020). Therefore, different consumption components offer insights into the vectors driving ingestive behaviors (Krakauer et al., 2017). However, interpreting the temporal organization of consumption remains challenging. Most studies on the encoding and salience of taste valence have been conducted in rats, and only a few have been performed in mice, an animal model with advanced genetic tools (Requejo-Mendoza et al., 2025). In this study, we demonstrated a custom-made setup that precisely records licking in freely behaving mice based on different experiences. We hypothesized that differential licking patterns encode the hedonic aspects of taste stimuli and are evolutionarily conserved. Next, we examined whether innate and learned aversive taste valences were dissociated. We conducted a comprehensive analysis that integrated the temporal domain of bursts, frequency domain of interlick intervals (ILIs), and multivariate analysis. Our findings reveal novel features of licking patterns that can be used in the future to better classify the emotional dimensions associated with taste and identify the biological mechanisms underlying the emotional tagging of taste experiences.

## Materials and methods

### Animals

Adult wild-type (WT; C57BL/6J) male mice (8–12 weeks old) were used in this study. The mice were kept in the local animal resource unit of the University of Haifa in a temperature-controlled environment under a 12-h dark / light cycle. Water and food pellets were provided *ad libitum*. All experiments and procedures were approved by the Animal Care and Use Committee (ethics licenses 283/13 and 518/18), as prescribed by the National Institutes of Health Guidelines for the Ethical Treatment of Animals.

### Experimental methods

#### Licking behavior apparatus

**FIGURE 1:**
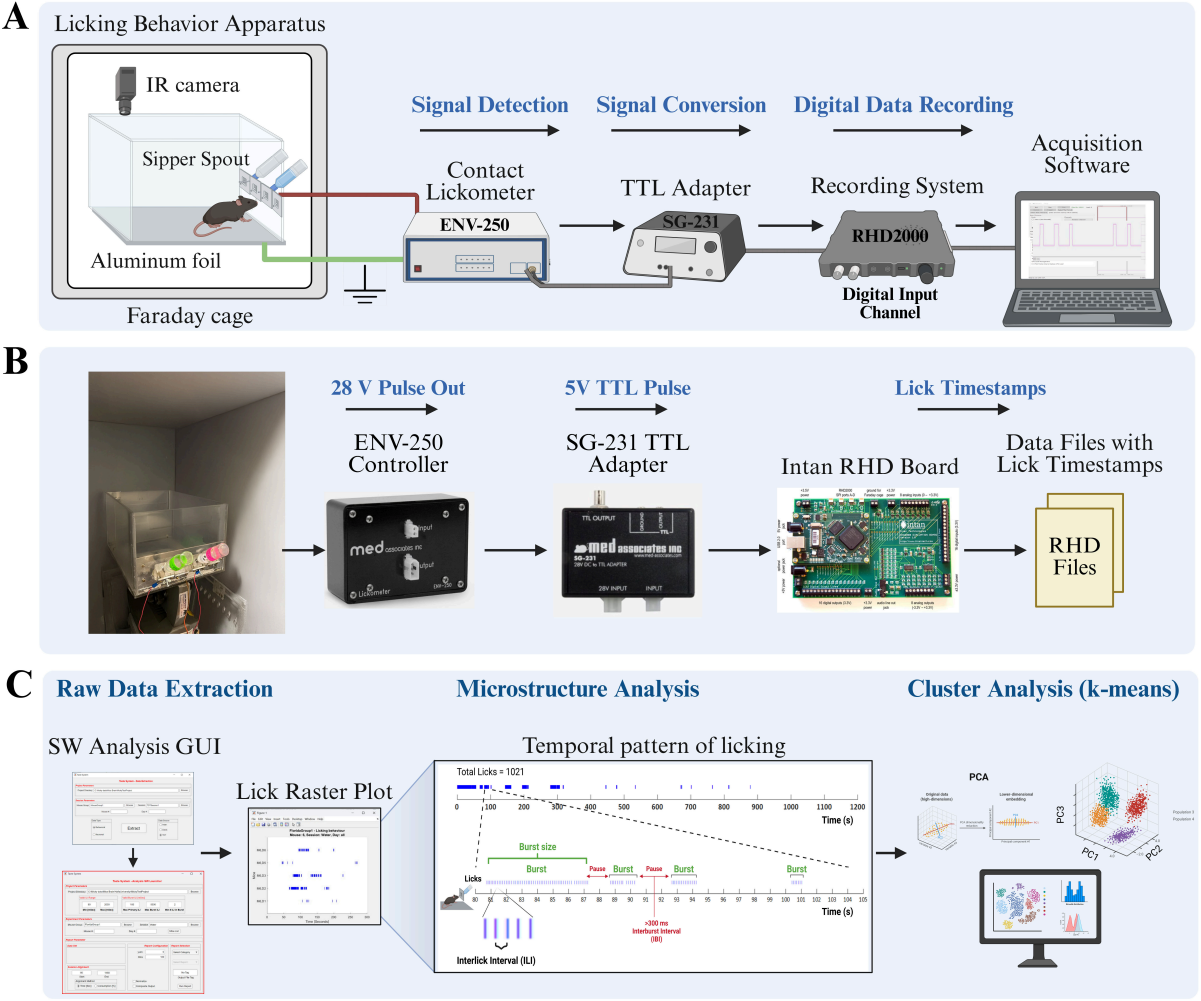
Custom-built lickometer system for high-resolution recording and analysis of voluntary licking behavior. **(A)** Schematic representation of the behavioral apparatus and data acquisition workflow. The mouse housed in a custom chamber located inside a grounded Faraday cage. An overhead infrared (IR) camera enables video monitoring. Licks are detected when the animal’s tongue makes contact with the metal sipper spout, thereby completing an electrical circuit with the conductive aluminum foil floor. A contact lickometer controller detects this event and generates a digital pulse. This pulse is subsequently converted into a standard 5V TTL signal using an adapter. These TTL pulses, each representing a single lick, are then transmitted to a data recording system and stored using the acquisition software. **(B)** Hardware components for the signal conversion and recording. The configuration illustrates the signal pathway from the lickometer controller (MED-SG-231) to the TTL adapter (SG-231) and, subsequently, to the digital data recording board (Intan RHD2000). This system digitizes licking events into raw data files that contain high-precision licking timestamps (RHD Files). **(C)** The Data analysis pipeline involves several key steps. Initially, raw data containing lick timestamps are extracted utilizing a custom MATLAB-based graphical user interface (GUI). Subsequently, microstructural analysis is conducted to characterize the temporal patterns of licking, which are visualized in lick raster plots and quantified through measures such as lick bursts and interlick intervals (ILIs). Ultimately, multivariate k-means cluster analysis is employed to classify distinct licking patterns based on their microstructural features. Figures are created by using BioRender (66587182).

Licking behavior was assessed using a custom-built apparatus, the schematic of which is shown in Figure 1. A complete Bill of Materials, including all commercial custom components required for construction, is provided in Supplementary Table 1.

For each experiment, a mouse was individually placed in a transparent acrylic chamber (30 L × 14.5 W × 16 H cm). To minimize environmental and electrical distractions, the chamber was situated within a sound-attenuating wooden cabinet lined with grounded metal sheets, functioning as a Faraday cage. A sheet of conductive aluminum foil covering the chamber floor served as the ground for the licking detection circuit. The behavior of the subjects was monitored throughout the experiment using an infrared (IR) camera. Liquid tastants were delivered through glass sipper bottles (MED-DAV-250BT-M, Med Associates Inc.) equipped with metal spouts mounted on the front wall of the chamber. Each sipper tube was centered within a lick access slot (2.6 H × 0.8 W cm) to allow for natural drinking posture.

#### Lick detection and data acquisition

A schematic representation of the data acquisition workflow is shown in Figure 1. Individual licking events were recorded using a high-sensitivity contact lickometer system. The system operates on the principle of circuit completion: a lick is registered when the subject contacts a metal sipper spout while standing on a grounded conductive floor. The process of detecting and recording licks was as follows:

##### Circuit connection

The core of the detection system consisted of two single-channel contact lickometer controllers (MED-ENV-250, Med Associates Inc.) dedicated to each sipper spout. An electrical wire connected the aluminum foil floor to the “Ground” terminal of each controller, creating a common ground. Another wire connected each metal sipper spout to the “Operate” (input) terminal of its respective controller. This two-controller configuration allowed for the independent and simultaneous recording of licking behavior from both bottles. This configuration ensured that the current passing through the animal was negligible (< 0.3 μA, as per manufacturer specifications).

##### Signal processing

Upon detecting a lick, the corresponding ENV-250 controller generated a 28V DC digital pulse. This signal was then sent to a 28V DC to TTL adapter (MED-SG-231, Med Associates Inc.), which converted the 28V pulse into a standard 5V Transistor-Transistor Logic (TTL) signal. The system was powered by a dedicated 28V DC power supply (MED-SG-501A, Med Associates Inc.).

##### Data recording

The resulting 5V TTL pulses, each representing a discrete timestamped lick, were sent to the digital input channels of an Intan RHD2000 evaluation system (Intan Technologies). The system recorded each digital event with a high temporal resolution, generating a data file with precise timing of all lick instances. This high-resolution data is suitable for subsequent detailed microstructural analysis of licking behavior, such as assessments of lick frequency, burst characteristics, and interlick intervals.

### Experimental procedures

*Ad libitum* water was removed from the mouse cages approximately 23 h before the test to increase motivation. The mice were randomly allocated to experimental groups. The group size range estimation was based on previously published methods and power calculations.^1^ For all experiments in this study, mice were water-deprived for 24 h and given water during a 20-min single-bottle session for three successive days to habituate them to water consumption and the experimental context. The allocation of animals, specific procedures, and timelines for each experiment are presented in Table 1.

**TABLE 1:** Summary of experimental groups and procedures.

Figure	Group	N	Technique	Treatment	Stimulus	Testing timeline
Figure 2	Water group 1	10	Single-bottle test		Water	4 Days
	Water group 2	10	Single-bottle test		Water	4 Days
	Water group 3	10	Single-bottle test		Water	4 Days
	Water group 4	10	Single-bottle test		Water	4 Days
	**Total**	**40**				
Figure 3	Test group	10	Single-bottle test		Water	3 Days
			Single-bottle test		Saccharin	1 Day
			Single-bottle test		Quinine	1 Day
Figure 4	CTA	10	Single-bottle test	0.24M LiCl (i.p)	Saccharin	7 Days
	CTA—control	10	Single-bottle test	Saline (i.p)	Saccharin	7 Days
Figure 5	CTA (learned aversive)	10	Single-bottle test	0.24M LiCl (i.p)	Saccharin	7 Days
	Quinine (innate aversive)	10	Single-bottle test		Quinine	4 Days

#### Recording of licking responses of water, saccharin, or quinine sessions

After three training days with water, the mice were presented with novel 0.5% saccharin during a 20-min recording session on the fourth day. The following day, the mice were given *ad libitum* access to water for one day. The mice were then deprived of water for 24 h. All mice were presented with novel 0.04% quinine during a 20-min recording session on the sixth day.

#### Conditioned taste aversion acquisition and licking response recording during CTA retrieval for saccharin

After three training days with water, the mice were presented with novel 0.5% saccharin during a 20-min recording session on the 4th day. 40 min after the start of the 20-min recording session (interstimulus interval, ISI = 40 min), mice were intraperitoneally injected with a 2% body weight dose of the malaise-inducing agent LiCl (0.14 M) (CTA group) as well as the unconditioned stimulus (US), while control animals received a weight-adjusted dose of saline (1% body weight) (CTA-control group). All mice were given water for 2 days during a 20-min single-bottle recording session. On the seventh day (retrieval day), all mice were tested during a 20-min session for saccharin retrieval.

### Data analysis

#### Acquisition and analysis of behavioral data

The Intan-recorded data were organized in a proprietary format. Each file recorded 1 min of activity, and the recorded data consisted of digitized samples of the recorded signal; for instance, for each data sample, a digital channel would show a logical “zero” or “one” value, and an analog signal would show the output of the A/D converter. At the sample rate of 20 K that we used, the lickometer data (for example) were sampled every 50 μs, and the recorded data included 20,000 logical values per second.

We used the RHD file reader provided by Intan to convert the recorded data into MATLAB arrays. Subsequently, we developed custom MATLAB routines to identify mouse clicks within a sequence of logical “zeros” and “ones” in the lickometer output signal. This process enabled us to generate a single MATLAB file containing a list of licking time stamps.

#### Analysis software

We developed a second MATLAB program that performed the following functions:

• All data files were organized in a rigidly defined folder hierarchy, enabling easy access to and management of data.
• We analyzed the relationships between the interlick intervals (ILIs) and generated a collection of derived information (lick count, burst count, burst length, burst efficiency, and histograms of ILI distributions) that could later be used to characterize mouse behavior as a proxy for its emotional and mental states.
• A set of graphs is generated to enable researchers to visually validate and develop a feel for the generated data (e.g., activity raster plots that show the licks on a time scale and ILI distribution graphs that show the licks on a frequency scale).
• Automated procedures for exporting the derived data into CSV files for further statistical analysis.

#### Burst analysis

Data files consisting of the onset times of each lick during the test session interlick intervals (ILIs) were analyzed using a custom-made MATLAB graphical user interface (GUI) to extract several microstructural variables. ILIs of < 60 ms were considered physiologically implausible and likely due to momentary loss of contact with the spout during an ongoing lick. The total number of licks was calculated as the total number of licks during the test session. One mouse licked less than 50 times on day three and was subsequently removed from the recording session.

#### ILI distribution histograms

Typically, mice lick in bursts (or bouts), with the number of licks per burst and the pauses between bursts being highly variable. While constructing a histogram of ILIs, we were interested in analyzing relatively short ILIs; therefore, we ignored all ILIs longer than 1000 ms. We also ignored ILIs shorter than 60 ms (owing to artifacts when the mouse touched the sipper tube; these intervals constituted less than 1% of the total number of intervals). We defined (N) as the total number of ILIs (i.e., the total number of licks minus one) collected during each 90-min recording session.

#### Bursts and pause duration

We defined a burst as three or more licks with ILIs of < 300 ms. This criterion was chosen based on the histogram distributions of the ILIs from the recordings of all sessions. Although alternative criteria, such as the 500 ms threshold commonly employed in studies of licking behavior, were considered, the 300 ms value was chosen because it most accurately reflected the natural break in our mouse ILI distribution and offered the most robust differentiation between our experimental conditions. Most ILIs were in the range of 60–300 ms, including approximately 90% of the ILIs. Using this criterion, the number of bursts (mean burst count) and average burst size (measured as licks per burst) were calculated for each mouse. ILIs > 300 ms were considered interburst intervals (pause durations).

#### Mean primary ILIs

The mean primary rate was calculated as the mean rate of all ILIs within the 60–180 ms range. This cutoff value (180 ms) was chosen to capture most of the licks (∼90%).

#### Lick efficiency

The lick efficiency was calculated as the proportion of primary ILIs within a burst to the total number of ILIs in the burst.

#### Percentage of ILIs within peaks

The percentage of ILIs at each peak was calculated. The first peak included ILIs in the 60–180 ms range, the second peak in the 180–300 ms range, and the third peak included ILIs > 300 ms.

#### Cumulative licks

The number of licks during each minute of the 20-min session was calculated for each mouse and converted to cumulative percentages of total licks that began in the first minute and ended at 100% consumption.

#### Relative frequency

The relative frequencies of ILI durations (60 ≤ ILIs ≤ 500 ms) were extracted from the data. The ILI distribution of all mice was averaged to obtain the mean ILI distribution for each group. The distribution was fitted to a bimodal or trimodal Gaussian using the MATLAB R2020b fitting function (*fit*). The calculated fitting curve overlapped with the relative frequency plots of the ILI distribution. The parameters (μ and σ) describe the theoretical fitting curves.

#### Principal component analysis

To reduce the dimensionality of the multivariable licking data, we performed Principal Component Analysis (PCA) using MATLAB R2020b and GraphPad Prism 9. For each analysis, PCA was conducted on the covariance matrix of the input variables. We used a covariance matrix instead of a correlation matrix for this study, despite the differing scales of our input variables (e.g., lick counts, burst sizes, and interlick interval durations). The covariance matrix preserves this inherent variance, ensuring that variables exhibiting larger fluctuations have a proportionally greater influence on principal components. The adequacy of the data for PCA was confirmed before the analysis. The Kaiser-Meyer-Olkin (KMO) measure of sampling adequacy was 0.65, indicating that the strength of the relationships among the variables was adequate. Additionally, Bartlett’s test of sphericity was statistically significant (*p* < 0.001) for each dataset, confirming that the variables were sufficiently inter-correlated to warrant the application of PCA (Budaev, 2010). The specific test statistics for each analysis are reported in the corresponding results sections (Supplementary Figures 3–5). The optimal number of principal components to retain was determined primarily using Parallel Analysis, a robust simulation-based method (Budaev, 2010), with a secondary check to ensure that the retained components explained more than 75% of the total variance of the original dataset. To facilitate interpretation, the retained components were subjected to an oblique Promax rotation, which allows the resulting components to be correlated (The Analysis Factor, 2012), which is a more biologically plausible assumption for interrelated neurobiological constructs (Johnson, 2018). A loading value of ≥ |0.70| was used as the threshold to identify a variable as a significant contributor to a component. To validate the model, we calculated the communality values for each variable to ensure that the solution adequately captured their variance (Budaev, 2010). Finally, to quantitatively assess the separation between experimental groups observed in the PCA plots, we applied a *post-hoc* k-means clustering algorithm to the subjects’ component scores, using the percentage of correctly classified subjects as an objective measure of the model’s discriminatory power.

### Statistical analysis

All statistical analyses were performed using SPSS, GraphPad Prism, and MATLAB R2020b. All data are presented as mean ± SEM unless otherwise specified. Statistical significance was set at *p* < 0.05. Non-parametric tests were selected because the sample sizes were too small to formally test for or assume a normal distribution, which is a key requirement for parametric tests. This approach is considered more rigorous for this type of data because it avoids making assumptions regarding the underlying data distribution. Specific tests were selected based on the experimental design as follows.

**FIGURE 2:**
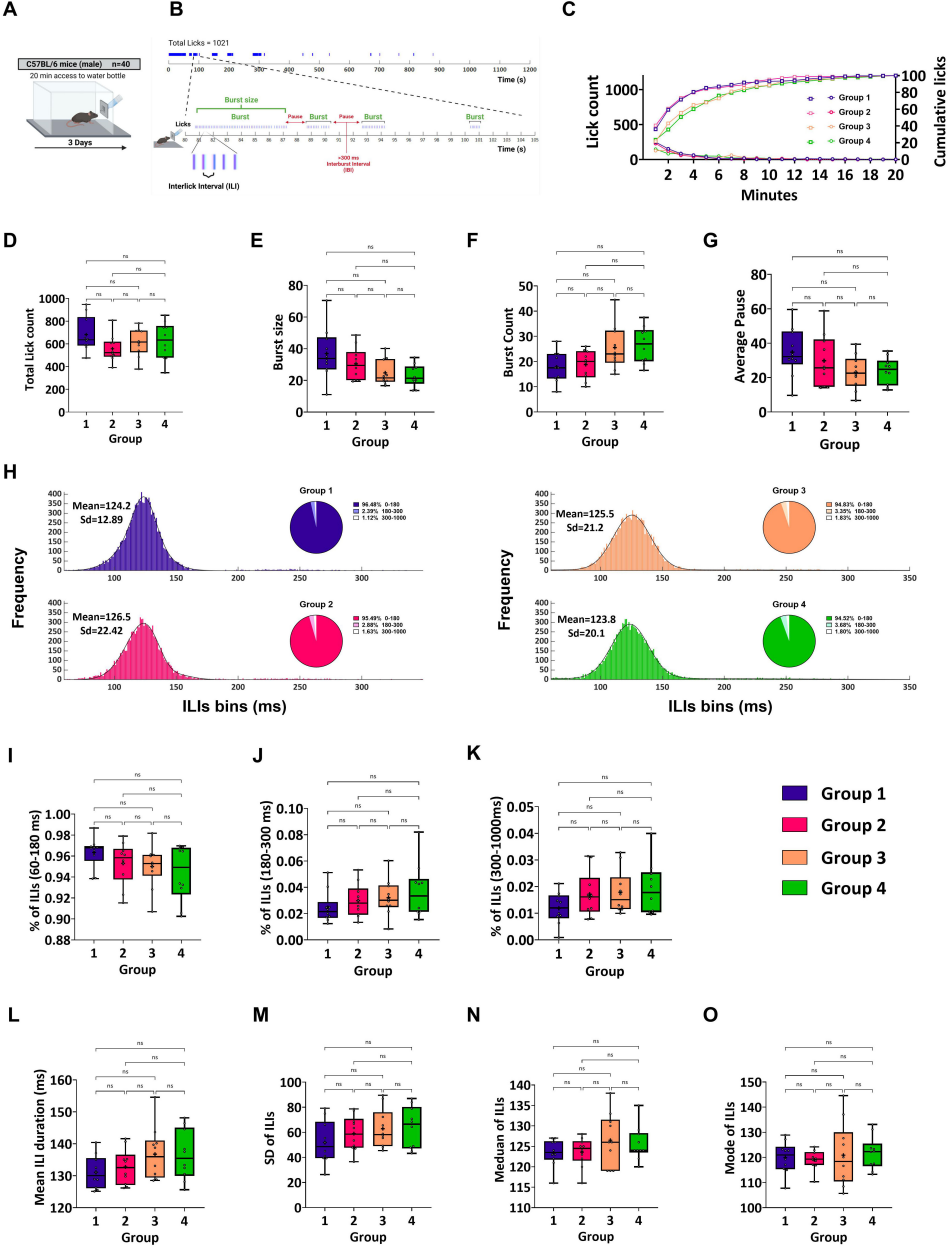
*In vivo* system validation and licking behavior assessment demonstrated the capability of the system to detect licks with high temporal resolution and accuracy. **(A)** Schematic representation of experimental design. All sessions were 20 min long. During the training phase, the mice were provided with water for 3 days. **(B)** (Top) Schematic illustration of the temporal pattern of licking in a 20-min session by an individual C57Bl/6J mouse during the water day. This mouse licked 1,021 times, which were organized within bursts of drinking. (Bottom) Expanded view of a 25-s section showing the temporal pattern of the licks. Vertical lines represent individual licks, and the intervals between them are defined as interlick intervals (ILIs). Bursts are runs of licks separated by interlick intervals (ILIs) ≤ 300 ms. **(C)** Cumulative mean licks (per 1-min bin) expressed as the percentage of total licks (right y-axis, 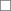) and rate of licking (left y-axis, 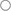) for Group 1 (purple), Group 2 (pink), Group 3 (light orange), and Group 4 (green) during a 20-min session. **(D)** Mean total lick count during a 20-min acquisition session. The mice licked in the same way in all groups. Group 1 (682.2 ± 48.67), Group 2 (558.2 ± 36.57), Group 3 (615.9 ± 39.21), Group 4 (625.7 ± 51.94). **(E)** Mean burst size. The burst sizes did not differ among the groups. Group 1 (36.94 ± 5.09), Group 2 (30.44 ± 3.22), Group 3 (24.75 ± 2.55) and Group 4 (23.01 ± 2.21). **(F**) Mean burst count. The mean burst counts were the same for all groups. Group 1 (17.85 ± 1.91), Group 2 (18.8 ± 1.78), Group 3 (25.55 ± 2.8) and Group 4 (26.6 ± 2.27). **(G)** Mean Average pause. The mean average pauses for all the groups were the same. Group 1 (34.81 ± 4.5), Group 2 (29.79 ± 4.77), Group 3 (22.99 ± 3.15) and Group 4 (23.90 ± 2.48). **(H)** Mean frequency distributions of ILIs (bin size = 5 ms) < 500 ms for each group. The distributions for all groups were fitted to a Gaussian curve model (Group 1, purple; Group 2, pink; Group 3, light orange; and Group 4, green). The primary peak of distributions is in the range of 60–180 ms (Group 1: μ1 = 124, σ1 = 12.89; Group 2: μ1 = 126.5, σ1 = 22.42; Group 3: μ1 = 125.5, σ1 = 21.2; Group 4: μ1 = 123.8, σ1 = 20.1). A pie chart is shown for each distribution with the percentage of ILIs within 60–180 ms, 180–300 ms, and 300 ms < ILIs < 1,000 ms. No differences were found between the groups in terms of ILI distribution or frequency of ILI per range (shown in the pie chart). **(I)** Percentage of total ILIs in the first peak (60–180 ms). All groups showed similar ILI between 60 and 180 ms frequency (Group 1: 96.34 ± 0.46%; Group 2: 95.30 ± 0.64%; Group 3: 95 ± 0.64%; Group 4: 94.39 ± 0.84%). **(J)** A similar percentage of total ILIs between 180 and 300 ms between the groups. In the second peak, the percentages of ILIs for Groups 1 (2.4 ± 0.38%), 2 (2.9 ± 0.39%), 3 (3.21 ± 0.44%), and 4 (3.7 ± 0.65%) are shown. **(K)** Percentage of ILIs in the third peak (300–1,000 ms). **(L)** The mean ILI duration was similar between the groups. Group 1 (131.1 ± 1.63), Group 2 (132.7 ± 1.77), Group 3 (136.8 ± 2.63), Group 4 (136.5 ± 2.48). **(M)** The mean Standard Deviation of ILIs was similar between the groups. Group 1 (51.88 ± 5.39), Group 2 (58.91 ± 4.45), Group 3 (62.85 ± 4.94), Group 4 (63.82 ± 5.26). **(N)** The mean and median ILIs were similar between the groups. Group 1 (123.5 ± 1.07), Group 2 (123.6 ± 1.13), Group 3 (126.5 ± 2.06), Group 4 (125.6 ± 1.33). **(O)** The mean mode of ILIs was similar between the groups. Group 1 (119.9 ± 2), Group 2 (119 ± 1.23), Group 3 (120.9 ± 4.03), Group 4 (121.8 ± 2.08). Data are shown as mean ± S.E.M. *p < 0.05, **p < 0.01, ***p<0.001, ****p < 0.0001.

For comparisons between three or more independent groups, such as the analysis of the four water groups shown in Figure 2, the Kruskal-Wallis test was performed. When this test yielded a significant result, Dunn’s multiple comparisons test was performed as a *post-hoc* analysis to identify specific differences between group pairs.

**FIGURE 3:**
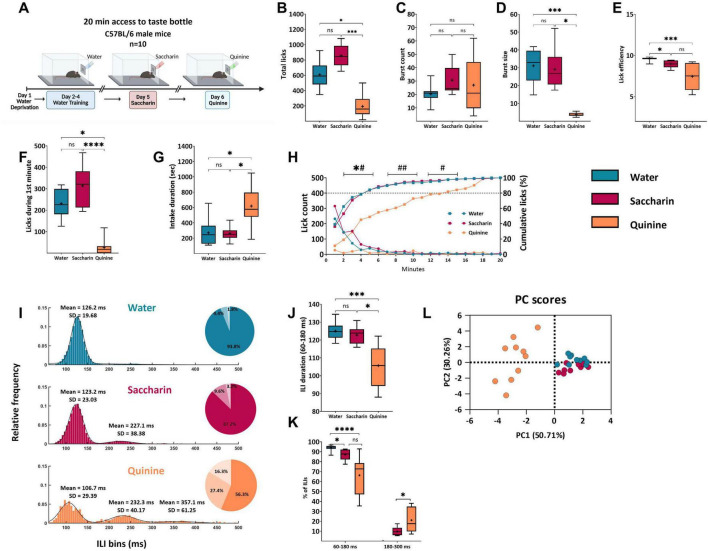
Licking microstructure reveals temporal and qualitative differences dependent on taste and valence. **(A)** Experimental design. Each session lasted for 20 min. The training phase commenced with 3 days of water administration to the mice, followed by tastant administration on subsequent days. **(B)** Mean total lick count during a 20-min acquisition session. Mice licked significantly less quinine (194.6 ± 44.61) than saccharin (857.9 ± 44.9) and water (609.9 ± 54.1). **(C)** Mean burst count. The mean burst counts for saccharin (30.75 ± 3.48), water (20.4 ± 2.1), and quinine (27 ± 6.2) did not differ between sessions. **(D)** Mean burst size. The size of the bursts during the quinine (3.83 ± 0.36) session was significantly shorter than that during other sessions. The saccharin burst size (29.18 ± 3.32) did not differ from that of water (31.12 ± 2.94) sessions. **(E)** Mean lick efficiency. Mice had higher lick efficiency during the water session (9.58 ± 0.07) than during the saccharin (8.97 ± 0.14) and quinine sessions (7.46 ± 0.48). **(F)** Mean lick count during the first minute of the session. Mice licked significantly less quinine (26.30 ± 11.62) than water (231.3 ± 20.71) and saccharin (314.1 ± 29.67). **(G)** Mean intake duration. The consumption time of 80% intake for the quinine session (619.3 ± 80.69 s) was longer than that for water (271.2 ± 54.38 s) or saccharin sessions (262.6 ± 28.18 s). **(H)** Rate of licking (left y-axis, 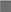) and cumulative mean licks expressed as a percentage of total licks (right y-axis, 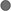) during 20-minute sessions with water (blue), saccharin (magenta), or quinine (orange). Data are shown as mean ± S.E.M. Statistical significance for comparisons against the quinine group is indicated. For the lick count analysis (0–5 min): ^*#^*p* < 0.05. For the cumulative intake analysis (5–15 min): ^#^*p* < 0.05, ^##^*p* < 0.01. **(I)** Mean frequency distributions of ILIs (bin size = 5 ms) < 500 ms for each taste. The water (blue) distribution was fitted to a Gaussian curve, saccharin (magenta) to a bimodal Gaussian curve, and quinine (orange) to a trimodal Gaussian curve. The primary peak of the distribution was in the range 60–180 ms (water: μ_1_ = 126.2, σ_1_ = 19.68; saccharin: μ_1_ = 123.2, σ_1_ = 23.03; quinine: μ_1_ = 106.7, σ_1_ = 29.39), and the second peak was approximately twice the height of the primary peak (saccharin: μ_2_ = 227.1, σ_2_ = 38.38; quinine: μ_2_ = 232.3, σ_2_ = 40.17). A third peak for quinine was observed for ILIs > 300 ms (quinine: μ_3_ = 357.1, σ_3_ = 61.25). A pie chart is shown for every distribution, with the percentage of ILIs within 60–180 ms, 180–300 ms, and > 300 ms. **(J)** Mean duration of primary ILIs Mean ILIs in the range of 60–180 ms were significantly shorter in the quinine session (105.7 ± 3.56 ms) than in the water (125 ± 1.63 ms) and saccharin sessions (122.7 ± 1.55 ms). **(K)** Percentage of total ILIs in bursts (60–180 ms and 180–300 ms). A significantly higher percentage of ILIs in the range of 60–180 ms during the water session (93.83 ± 0.93%) compared to saccharin (87.1 ± 1.66%) and quinine (66.32 ± 5.98%). In the second peak (180–300 ms), there was a significantly higher percentage of ILIs during the quinine session (21.1 ± 3.81%) than during the saccharin session (9.75 ± 1.29%). (L) Cluster distribution along Dimensions 1 and 2 Principal component analysis (PCA) of the three tastants. The two-dimensional scatter plot shows the differences between appetitive (saccharin), aversive (quinine), and water sessions. Each color represents a single tastant, and each dot represents a mouse. Nine variables were used: lick count, burst count, burst size, primary rate, effective rate, ick efficiency, average ILI, standard ILI, and mean primary ILI. Data are shown as mean ± S.E.M. *p < 0.05, **p < 0.01, ***p < 0.001, ****p < 0.0001.

For repeated measures comparisons across three or more conditions within the same subjects, such as the analysis of responses to water, saccharin, and quinine (Figure 3), the Friedman test was applied. Significant results were followed by Dunn’s multiple comparisons test for subsequent pairwise comparisons.

**FIGURE 4:**
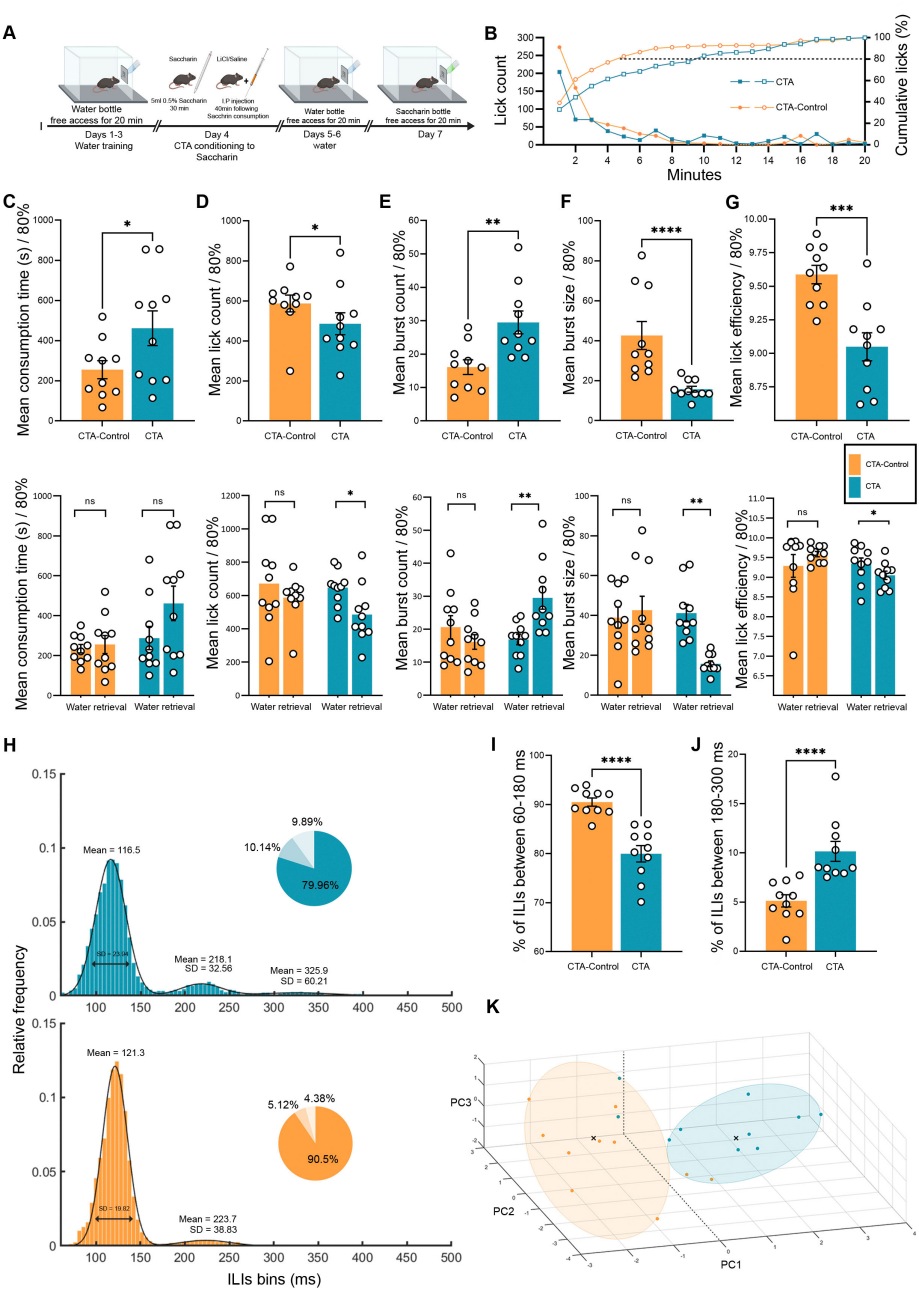
Licking microstructure reveals how mouse drinking behavior is altered by shifting the valence of the same taste. **(A)** Schematic of freely moving mice in the CTA test using the Lickometer paradigm. Twenty adult (8–12 weeks) WT mice were tested using a 7-day protocol to examine CTA retrieval. Mice were divided into CTA-control (orange, *n* = 10) and CTA (blue, *n* = 10) groups and placed under water restriction for 23 h before the water training days. The mice consumed either water or tastants during a 20-min session for the next 7 days. All mice were trained with water for 4 days. On day 5 (conditioning), the mice were administered 0.5% saccharin during the session, and 20 min later, they were injected intraperitoneally with either LiCl (CTA group) or saline (CTA-control group). The mice were then subjected to one water test session per day for the next 2 days. On the last day (retrieval day), all mice underwent a single saccharin session. **(B)** Cumulative mean licks are defined as the percentage of total licks during a 20-min saccharin retrieval session for the CTA (blue) and CTA control (orange) groups (right y-axis; 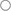, 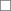, 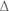). CTA-control mice consumed 80% of their total intake during saccharin retrieval faster (∼5 min) than CTA mice (∼10 min). Mean lick count progression for the CTA and CTA-control groups (left y-axis; 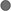, 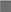, 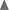). Figs. C-G (Top) Comparison of licking microstructure parameters during CTA retrieval for saccharin in the CTA and CTA-control mice. (Bottom) Comparison of water and quinine retrieval sessions in CTA and CTA-control mice (80% consumption). **(C)** Mean intake duration. (Top) The consumption time of 80% intake for CTA mice was significantly longer (461.82 ± 86.39 s) than that for CTA-control mice (255.46 ± 44.88 s) during the retrieval session. (Bottom) There were no significant differences in either CTA mice (water day: 287.18 ± 57.91 s) or CTA control (water day: 234.14 ± 21.23 s) on the retrieval day compared to the water day. **(D)** Mean lick count. (Top) CTA-control mice licked significantly more (587.10 ± 42.05) than CTA mice (485.90 ± 54.90). (Bottom) CTA mice showed a significant reduction in the total number of licks from the water day (646.50 ± 32.36) on the test day, while there were no differences in CTA-control mice (water day: 671.60 ± 84.28). **(E)** Mean burst count. (Top) CTA mice displayed significantly more bursts (29.50 ± 3.41) than the CTA control mice (16.10 ± 2.20). (Bottom) CTA mice showed a significant increase in the number of bursts from the water day (16.90 ± 1.59) to the retrieval day (as compared with unchanged burst count in CTA-control mice (water day: 20.60 ± 3.50). **(F)** Mean burst size. (Top) CTA-Control mice consumed saccharin in significantly larger bursts of licking (42.61 ± 7.00) than did CTA mice (15.70 ± 1.47) on the CTA retrieval day. (Bottom) CTA mice showed a significant reduction in burst size from water day (41.20 ± 4.35) to retrieval day compared with the unchanged burst size in CTA-control mice (water day: 38.86 ± 5.30). **(G)** Total number of licks during the first 3 min. The lick count for the CTA group (345.40 ± 51.61) was significantly lower than that of the CTA-control group (501.50 ± 45.12). The lick count for CTA mice during CTA retrieval for saccharin was lower than that for water (520.40 ± 47.89), whereas there was no significant difference in the CTA-Control group (526.00 ± 36.80). **(H)** Mean frequency distributions of ILIs (5 ms bins) for CTA and CTA-control mice on the saccharin retrieval day. The distribution was fitted to a bimodal Gaussian curve for the CTA control and a trimodal Gaussian curve for the CTA. The primary peak for both groups was in the range 60–180 ms (CTA: μ1 = 116.50, σ1 = 23.94; CTA-control: μ1 = 121.30, σ1 = 19.82), whereas that for both groups was in the range 180–300 ms (CTA: μ2 = 218.10, σ2 = 32.56; CTA-control: μ2 = 223.70, σ2 = 38.83). The distribution in the CTA group showed a third peak (μ3 = 325.90, σ3 = 60.21). For every distribution, a pie chart shows the percentage of ILIs within 60–180, 180–300, and >300 ms. **(I)** Mean percentage of ILIs in the first peak (60–180 ms). The percentage of ILIs in the range of 60–180 was significantly higher for the CTA-control (90.50 ± 0.84%) than for the CTA group (79.96 ± 1.67%). **(J)** Mean percentage of ILIs in the second peak (180–300 ms). The percentage of ILIs in the second peak was significantly higher in CTA mice (10.14 ± 1.01%) than in CTA-control mice (5.12 ± 0.62%). **(K)** Principal component analysis (PCA) of CTA and CTA controls. A three-dimensional scatter plot of the PCA shows the separation of different data from the CTA and control groups. Each color represents a single group, and each circle represents a cluster detected using the k-means algorithm (*k* = 2). Nine variables were used: total licks, burst count, burst size, pick efficiency, primary rate, effective rate, pause duration, intake duration, and licks during the first 3 min. Data are shown as mean ± S.E.M. *p < 0.05, **p < 0.01, ***p < 0.001, ****p < 0.0001.

**FIGURE 5:**
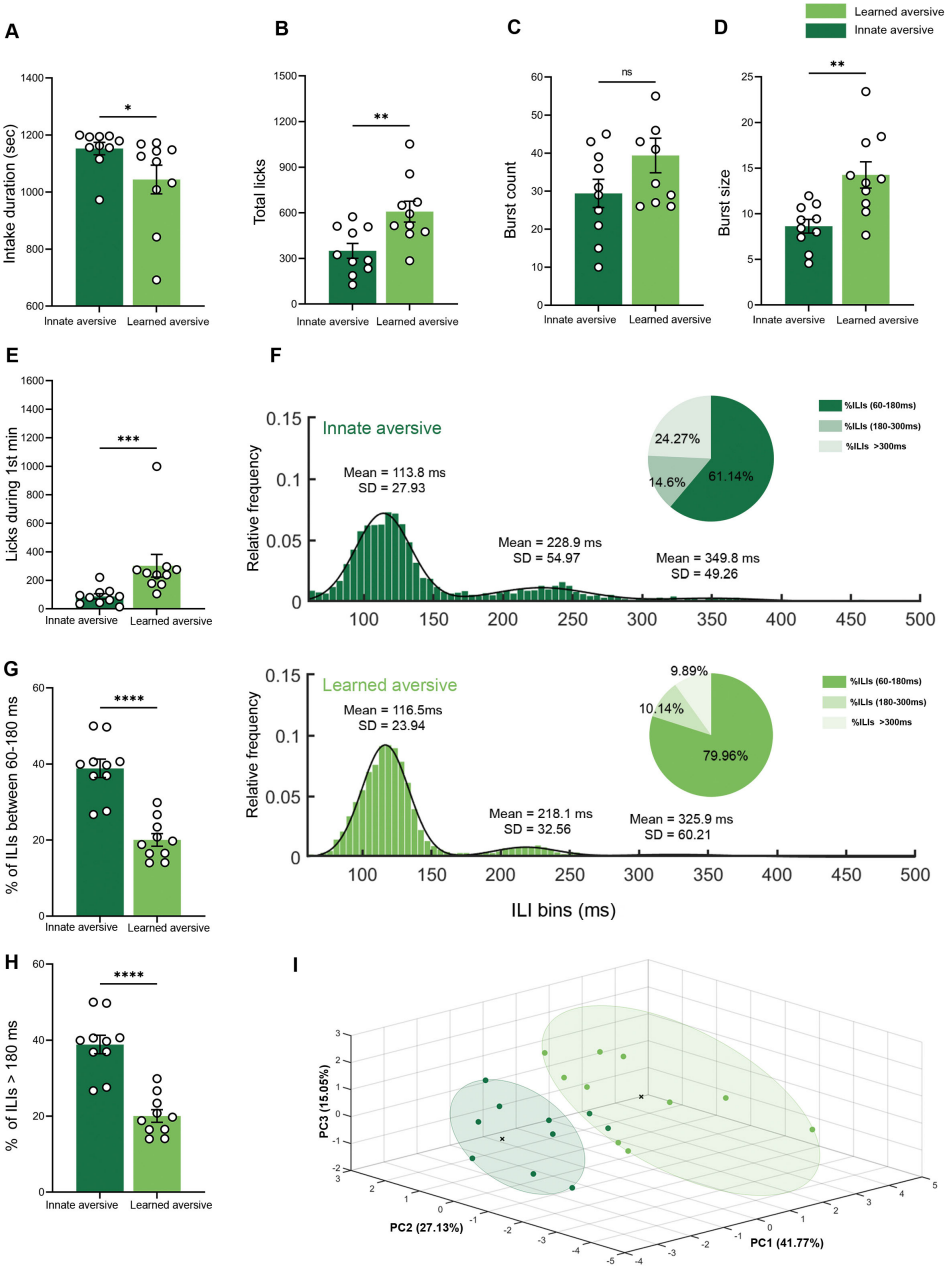
Innate and learned aversive tastants showing different licking microstructural patterns. **(A)** Mean intake duration. The consumption time for the total intake of CTA mice was lower (461.82 ± 86.38 s) during the retrieval session than during the quinine session (1152.53 ± 21.62 s). **(B)** Mean lick count. The CTA group licked significantly more (485.00 ± 54.90) than the quinine group (350.00 ± 48.80). **(C)** Mean burst count. No significant differences were observed between the CTA (39.40 ± 4.54) and quinine (29.40 ± 3.71) groups. **(D)** Mean burst size. The burst size during the quinine (8.63 ± 0.75) session was shorter than that during saccharin session in the CTA (15.70 ± 1.47) group. **(E)** Mean lick count during the first minute of the session. The CTA group licked significantly more (225.11 ± 20.56) than the quinine group (87.40 ± 18.43). **(F)** Mean frequency distributions of ILIs (5 ms bins) for innate and learned aversive tastants. The distribution was fitted to a trimodal Gaussian curve for both distributions. The primary peak for both groups was in the range of 60–180 ms (CTA: μ1 = 116.50, σ1 = 23.94; quinine: μ_1_ = 113.90, σ_1_ = 28.08), and the secondary peak for both groups was in the range of 180–300 ms (CTA: μ2 = 218.10, σ2 = 32.56; quinine: μ_2_ = 227.70, σ_2_ = 48.84). The distribution of the CTA group showed a third peak (CTA: μ3 = 325.90, σ3 = 60.21; quinine: μ_3_ = 349.80, σ_3_ = 49.26). A pie chart is shown for each distribution, with the percentage of ILIs within 60–180, 180–300, and >300 ms. **(G)** Mean percentage of ILIs in the first (60–180 ms) and second (180–300 ms) peaks. The percentage of ILIs in the range of 60–180 was significantly higher in the CTA (79.96 ± 1.67%) group than in the quinine (61.14 ± 2.43%) group. **(H)** Mean percentage of ILIs longer than 180 ms the percentage of ILIs longer than 180 ms was significantly lower for the CTA mice (20.04 ± 1.67%) than for quinine-treated (38.86 ± 2.43%) mice. **(I)** Principal Component Analysis (PCA) of innate and learned aversive tastants. A three-dimensional scatter plot of the PCA shows the separation of the different data from CTA and quinine. Each color represents a single group, and each circle represents a cluster detected by the k-means algorithm (*k* = 2). Nine variables were used: total licks, burst count, burst size, pick efficiency, primary rate, effective rate, pause duration, intake duration, and licks during the first 3 min. Data are shown as mean ± S.E.M. *p < 0.05, **p < 0.01, ***p < 0.001, ****p < 0.0001.

For comparisons between two independent groups, such as CTA vs. CTA-control (Figure 4) or innate aversive vs. learned aversive groups (Figure 5), the Mann-Whitney U test was used.

For comparisons between two paired or matched conditions within the same subjects, such as comparing licking behavior on a retrieval day to a water day for the same animal (Figure 4), the Wilcoxon matched-pairs signed-rank test was used.

To compare the entire data distributions, specifically for interlick intervals (ILIs), the two-sample Kolmogorov-Smirnov (KS) test was applied. A Bonferroni correction was applied to the *p*-value threshold to control for the family wise error rate when multiple KS test comparisons were performed.

For multivariate analysis, Principal Component Analysis (PCA) was used to reduce the dimensionality of the licking microstructure data and visualize the separation between experimental conditions. The suitability of the data for factor analysis was first confirmed using Bartlett’s test of sphericity. Parallel analysis was used to determine the number of principal components to be retained in the model. Finally, the k-means clustering algorithm was applied to the principal components to quantify the separability of the groups in a reduced dimensional space.

### Data analysis software and code accessibility

All behavioral data were processed and analyzed using TasteSystemSW, a custom software suite developed in MATLAB. This software comprises two primary modules: TasteExtractData, which facilitates the conversion of raw data from recording systems (Intan, TDT) into a usable format, and TasteAnalysisSW, which conducts microstructural analysis, including ILI distributions and PCA, and generates all the figures and reports presented in this study. The complete software package is accessible in the GitHub repository,^2^ and a comprehensive guide on installation and usage is provided in Supplementary File 1 (Software User Guide).

## Results

### *In vivo* behavioral validation demonstrated the setup’s capability to detect licks with high temporal resolution and precision

To investigate the reproducibility of the behavioral responses induced by the system, 40 naive water-restricted B6 mice were given water in a 20-min session for three consecutive days to habituate to the experimental context (Figure 2A). The experiment was performed to determine whether any changes in the behavior of the mice could be attributed to the water stimulus and whether similar results could be reproduced in different groups of mice. First, we analyzed offline time-dependent changes in the ingestive behavior of the four groups of mice using a custom-made software analysis program (TasteAnalysisSW; see Methods) that we developed (Figure 1). For all experiments in this study, licks were organized into a few discrete bursts of drinking behavior, interrupted by pauses. Data analysis was performed individually for each mouse. Next, the values were averaged across all the members of the same group. For example, a single C57Bl/6J mouse licked 1021 times during the water session (Figure 2B). We hypothesized that mice within groups would exhibit similar licking patterns in both the time and frequency domains. For this study, we defined a burst as three or more licks with an ILIs < 300 ms (see Methods). Data from two water sessions in four independent groups of mice were analyzed to validate the consistency and reliability of the behavioral recordings obtained using this system. The intake progression for mice in each group over the 20-min session, as indicated by the mean number of licks per minute, showed that licking declined within the first 4 min, probably because of the satiety effect, and stabilized after 10 min (Figure 2C, left y-axis). Intake in the water sessions was very similar for Groups 1 and 2, with mice from Groups 3 and 4 reducing their intake rate across the session faster than those in the other two groups. The cumulative probability curves showed that 80% of intake was completed within the first ∼4.5 min for mice from groups 1 and 2 and in ∼7 min for mice from groups 3 and 4 (Kruskal–Wallis test, U = 17.05, *p* = 0.0007; Dunn’s multiple comparisons tests: G1 vs. G2: *p* > 0.05, G1 vs. G3: *p* > 0.05, G1 vs. G4: *p* = 0.015, G2 vs. G3: *p* = 0.045, G2 vs. G4: *p* = 0.003, G3 vs. G4: *p* > 0.05, Figure 2C, right y-axis). No statistical differences were found among all groups for any of the other microstructural parameters that were analyzed for the water sessions (total lick count: Kruskal–Wallis test, U = 3.328; burst count: Kruskal–Wallis test, U = 8.729; burst size: Kruskal–Wallis test, U = 7.087; average pause: Kruskal–Wallis test, U = 4.913, all *p* > 0.05, Figures 2D–G). Next, we analyzed the changes in the mean licking frequency of the four groups of mice during the 20-min water session. Frequency histograms (binned at 5 ms) were generated using 60 ms ≤ ILIs ≤ 1,000 ms. The Kolmogorov–Smirnov (KS) test revealed no significant differences between the groups (two-sample KS test with Bonferroni correction, p > 0.05 for all comparisons; Figure 2H). We compared the proportions and mean durations of the ILIs for three distribution ranges: 60–180, 180–300, and 300–1,000 ms. Indeed, most ILIs (>90%) within the bursts ranged from 60 to 180 ms and showed no significant differences between the groups (Kruskal–Wallis test, U = 4.702, *p* > 0.05, Figure 2I). In addition, the MPI was stable across the different groups (Figure 2J; see Methods). Therefore, we extended the statistical analysis to test the proportions and mean durations of the remaining ILI ranges. Accordingly, the proportion and mean duration of ILIs in the 180–300 ms and 300–1000 ms ranges showed no significant differences across the different groups (180–300 ms: Kruskal–Wallis test, U = 3.784; 300–1,000 ms: Kruskal–Wallis test, U = 3.956, all *p* > 0.05; Figures 2J,K). To determine the mode of distribution with relatively high temporal precision (1 ms resolution) and detect differences between groups on the order of a few milliseconds, histograms were binned at 1 ms to filter out fast variations. We calculated several statistical measures of the central tendency of the ILI distribution for each mouse tested during the water session. These parameters included the mean, standard deviation (SD), mode, and median values of ILIs. As shown in Figures 2L–O, no significant differences were observed between the groups (mean ILI duration: Kruskal–Wallis test, U = 3.841; SD of ILIs: Kruskal–Wallis test, U = 3.332; mode of ILIs: Kruskal–Wallis test, U = 0.7801; median of ILIs: Kruskal–Wallis test, U = 1.663; all *p* > 0.05; Figures 2L–O). These results are consistent with previously reported licking rates in C57Bl/6J mice (Johnson et al., 2010; Boughter et al., 2007).

### Taste valence is captured through multiple parameters measured in a novel, customized experimental setup in freely behaving mice

Previous studies have shown that, beyond measuring total intake, the licking microstructure in rodents provides more nuanced behavioral information about the parameters that control food intake, particularly stimulus evaluation and palatability (Dwyer, 2012; Gero, 2020; Naneix et al., 2020). Using our established setup, we aimed to identify these valence differences. For all experiments in this study, water-restricted mice were initially given water during a 20-min single-bottle session for three consecutive days to habituate them to the experimental environment. We analyzed several microstructural parameters for each consumption session, including total licks, licks during the 1st minute, burst count, burst size, lick efficiency, pause duration, intake duration, and interlick intervals (ILIs).

We tested the hypothesis that different parameters of the licking microstructure characterize innate appetitive or aversive tastants. To this end, we examined the licking behavior of water-restricted mice with novel 0.5% saccharin (appetitive, low metabolic value) and novel 0.04% quinine (aversive, low metabolic value) solutions during a 20-min single-bottle acquisition session on subsequent days. As expected, the total licks during the session decreased significantly during quinine consumption compared with those during water and saccharin consumption [Friedman’s test, χ^2^_(2)_ = 16.80, *p* < 0.0001; Dunn’s multiple comparisons tests: water vs. saccharin, *p* > 0.05; saccharin vs. quinine, *p* = 0.0002; water vs. quinine, *p* = 0.022; Figure 3B], consistent with the results from previous studies in rats (Hsiao and Fan, 1993; Spector and St John, 1998). Burst count reflects satiation and post-ingestive feedback (Dwyer, 2012; Gero, 2020; Johnson, 2018; Spector, 2015). There were no significant differences in burst counts among the three tastants [Friedman’s test χ2(2)=3.2,p>0.05; Dunn’s multiple comparisons test: all *p* > 0.05; Figure 3C]. In contrast, consistent with previous studies (Hsiao and Fan, 1993; Spector and St John, 1998), mice consumed quinine in significantly shorter bursts than during the saccharin and water sessions [Friedman’s test, χ^2^_(2)_ = 15.8; Dunn’s multiple comparison tests: *p* < 0.0001; water vs. saccharin: *p* > 0.05, saccharin vs. quinine: *p* = 0.011, water vs. quinine: *p* = 0.0004; Figure 3D]. These findings reinforce the general assumption that burst size reflects palatability, as saccharin and water have positive values in mice (Dwyer, 2012; Gero, 2020; Johnson, 2018; Spector, 2015). Mice licked more efficiently (see Methods) in the water session than in the saccharin or quinine sessions [Friedman’s test, χ^2^_(2)_ = 16.8, *p* < 0.0001; Dunn’s multiple comparison test: water vs. saccharin, *p* = 0.022; saccharin vs. quinine, *p* > 0.05; water vs. quinine, *p* = 0.0002; Figure 3E]. The number of licks produced during the first minute, a measure assumed to be strongly influenced by the stimulus’s orosensory properties and relatively unaffected by post-ingestive events (Davis and Perez, 1993; Davis and Smith, 1992), decreased significantly by approximately 60% when quinine was present compared to when water and saccharin were present [Friedman’s test, χ^2^_(2)_ = 18.20, *p* < 0.0001; Dunn’s multiple comparisons tests: water vs. saccharin: *p* > 0.05; saccharin vs. quinine: *p* < 0.0001; water vs. quinine: *p* = 0.041; Figure 3F].

As shown in the intake progression over the 20-min session (Figure 3H), the intake rate, indicated by the mean licks per min bin of quinine, was significantly lower than that of water or saccharin. Mice showed similar licking rates during the first 5 min of the water and saccharin sessions [Friedman’s test, χ^2^_(2)_ = 16.8, *p* < 0.0001; Dunn’s multiple comparisons tests: water vs. saccharin, *p* > 0.05; saccharin vs. quinine, *p* = 0.0002; water vs. quinine, *p* = 0.022; Figure 3H, left y-axis]. While a drop-in lick count characterized the session with saccharin and water after the first 5–6 min, probably because of satiety, the lick counts remained consistently low throughout the quinine session, as satiety remained low across the entirety of this session (Figure 3H, right y-axis). In addition, the cumulative intake of saccharin and water during the first 10 min was significantly different from that of quinine [0–5 min: Friedman’s test, χ^2^_(2)_ = 16.8, *p* < 0.0001; Dunn’s multiple comparisons test: water vs. saccharin: *p* > 0.05, saccharin vs. quinine: *p* = 0.0002, water vs. quinine: *p* = 0.022; 5–10 min: Friedman’s test, χ^2^_(2)_ = 15.8, *p* < 0.0001; Dunn’s multiple comparisons test: *p* > 0.05, saccharin vs. quinine: *p* = 0.011, water vs. quinine: *p* = 0.0004; Figure 3H]. Specifically, 80% of the fluid intake was consumed within the first 4–5 min of the water and saccharin sessions, while the consumption of quinine took approximately 12 min, as demonstrated by the cumulative probability curves [Friedman’s test, χ^2^_(2)_ = 9.8, *p* = 0.0063; Dunn’s multiple comparisons test: water vs. saccharin: *p* > 0.05, saccharin vs. quinine: *p* = 0.041, water vs. quinine: *p* = 0.011; Figure 3H right axes, Figure 3G]. As previously noted, when rodents were administered a spout containing a tastant, they consumed fluid at a consistent frequency of 6–7 Hz in rats (Davis and Smith, 1992) and 8–10 Hz in mice (Johnson et al., 2010; Boughter et al., 2007). The temporal distribution of licking pauses can distinguish between stimulatory and inhibitory influences on ingestive behaviors (Davis and Smith, 1992; Spector and St John, 1998). Interestingly, there are three distinct ILI distribution regions (Davis and Smith, 1992). In the first region, 90% of the ILIs exhibited a sharp peak with a steep decline on either side. This distribution is thought to be controlled by a central pattern generator (CPG) in the brainstem and reflects rhythmic tongue movements in the rodent (Wiesenfeld et al., 1977). A shift in the distribution of these ILIs within the burst indicates alterations in oromotor function, independent of palatability and post-ingestive effects (Swick et al., 2015). To test this hypothesis, we examined the distribution of ILIs generated by mice across all experimental sessions. The frequency distributions of ILIs < 500 ms (5 ms bins) were fitted to a Gaussian curve for water, bimodal Gaussian curve for saccharin, and trimodal Gaussian curve for quinine. We used the non-parametric KS test to compare the probability distribution of ILIs in each group to determine whether they originated from the same population. The KS test showed significant differences in the distribution of ILIs between quinine, water, and saccharin sessions, indicating that the ILIs from each group came from a differentially distributed population (two-sample KS test with Bonferroni correction: water vs. quinine: *p* < 0.0001, saccharin vs. quinine: *p* < 0.0001, water vs. quinine: *p* = 0.011; Figure 3I). To examine how the ILIs distribution differed for each group, we measured several statistical parameters characterizing the distribution of ILIs when mice drank water, saccharin, or quinine. The first peak accounted for the majority of licks (ILIs of 60–180 ms) when the total number of ILIs was < 1,000 ms. The other two peaks reflected ILIs of 180–300 ms and > 300 ms. The quinine ILI distribution showed a significantly shorter mean duration of ILIs within the first peak region compared to water and saccharin [Friedman’s test: χ2(2)=15.80,p<0.0001; Dunn’s multiple comparisons tests: water vs. saccharin: *p* > 0.05, saccharin vs. quinine: *p* < 0.011, water vs. quinine: *p* < 0.0004; Figure 3J]. Water showed a higher proportion of ILIs in the same region as quinine and saccharin [Friedman’s test: χ2(2)=18.20,p<0.0001; Dunn’s multiple comparisons test: water vs. saccharin: *p* = 0.0417, saccharin vs. quinine: *p* > 0.05, water vs. quinine: *p* < 0.0001; Figure 3K]. In contrast, quinine demonstrated a significantly higher percentage of ILIs between 180 and 300 ms (second peak) than saccharin (Wilcoxon matched-pairs signed-rank test, *W* = 41, *p* = 0.037; Figure 3J). The decrease in licking burst size, accompanied by an increase in the proportion of longer ILIs with quinine than with water and saccharin, indicates the scattering of licking responses and fragmentation of ingestive behavior. Taken together, these results indicate that the ILI distribution histograms show significant differences depending on the type of taste consumed. PCA, a dimensionality reduction method, was used to present and compare the cumulative contributions of the different parameters. Next, we applied the k-means algorithm for clustering, which searches for an optimal division of points into a predetermined number of clusters within an unlabeled, multidimensional dataset. The principal components of the data are shown in a scatter plot (*n* = 30; Figure 3L). Bartlett’s sphericity test was used to determine the adequacy of the correlation matrix (Bartlett’s sphericity test: χ^2^ = 459.70, df =36, *p* < 0.0001). After PCA, oblique factor rotation was performed, and the number of factors was chosen according to the explained proportion of the total variance (variance > 75%) and the height of the communality (91.5%) (Supplementary Figure 3A) (S. V. Budaev, 2010). We used parallel analysis, which revealed two principal components explaining 50.71% (PC1), 30.26% (PC2), and 10.50% (PC3) of the total variance, and collectively explained 91.48% of the variance between the different taste valences (Figure 3L; Supplementary Figure 3B). The different tastants showed a good degree of separation in the PCA space (k-means clustering, 86.6% correctly clustered elements). PC1 distinguished between aversive and appetitive (including water) tastants and was characterized mainly by negative loadings of SD ILIs and the MPI. It was positively correlated with the lick count, burst size, lick efficiency, and ILI duration in the range of 60–180 ms. PC2 was positively correlated with the effective rate and MPI and negatively correlated with the burst count (Supplementary Figure 3C).

### Licking microstructure analysis segregates the same taste with different valence through diverse parameters

We characterized the microstructural patterns of licking behavior in response to dissociated quinine (novel and innately aversive), saccharin (novel and innately appetitive), and water (Figure 3). To determine whether learned taste aversion affects behavior in a manner similar to innate aversion to quinine, we examined the licking microstructure after CTA retrieval (Figure 4). After 3 days of water training sessions to habituate the mice to the experimental setup (Figure 4A), the mice were subjected to a single 20-min session on the fourth day (CTA conditioning to saccharin) with novel saccharin in the lickometer rig, and 40 min after the beginning of the session, the mice were intraperitoneally injected with a 2% body weight dose of either LiCl (0.14 M, CTA group) or saline (CTA control group). During the next 2 days, all mice were given water. On the seventh day (retrieval day), the mice were administered saccharin for a 20-min session (Figure 4A). We examined the intake as a function of time for each group during the retrieval session, as represented by minute-by-minute accuracy (Figure 4B). The mean number of licks declined over the 20-min session (2-min bins) for the CTA and CTA control mice, with the greatest intake occurring in the first 5 min of the session, and then stabilized in the last 15 min at a lower consumption level. In the first 5 min, the CTA group showed a rapid decrease in water intake compared to that of the control group (Figure 4B, left y-axis). The cumulative mean licks as a percentage of the total licks for the saccharin retrieval session showed that saccharin consumption was slower in the CTA mice than in the CTA control mice. Further analyses were conducted on 80% of the intake, as satiation in the last quarter led to random licking behavior (Figure 4B, right y-axis). We compared the microstructural parameters between mice trained in the CTA and CTA controls during taste memory retrieval for saccharin (Figures 4C–G, top). In addition, for each group separately, we compared the microstructural parameters measured on the retrieval day with those measured on the water day (day 6) (Figures 4C–G, bottom). Indeed, CTA mice consumed 80% of the total intake significantly slower than the CTA control mice (independent sample Mann–Whitney test: *U* = 26, *p* < 0.05; *p* = 0.037; Figure 4C, top panel). In addition, CTA mice exhibited a trend of longer consumption time than their water session on day 6, although this effect was not significant. The CTA control mice did not show any difference in consumption duration between retrieval and water days (dependent samples Wilcoxon test, CTA: *Z* =–1.38, *p* = 0.084; CTA control: *Z* =–0.46, *p* = 0.323; Figure 4C, bottom). The mean number of total licks in the CTA group was significantly lower than that in the CTA control group on the retrieval day (independent samples Mann–Whitney test: *U* = 26, *p* = 0.037; Figure 4D top) and compared to the water day. No significant difference was observed in the total number of licks between the water and retrieval days in the CTA control group (dependent samples Wilcoxon test, CTA: *Z* =–1.99, *p* = 0.023; CTA control: *Z* =–1.07, *p* = 0.142; Figure 4D, bottom). During the retrieval day, CTA mice showed a significantly increased burst count for saccharin compared to CTA control mice (independent samples Mann–Whitney test: U = 13, *p* = 0.005; Figure 4E, top) compared to water. CTA control mice displayed no significant differences between retrieval and water days (dependent samples Wilcoxon test, CTA: *Z* =–2.66, *p* < 0.0001; CTA control: *Z* =–1.27, *p* = 0.101; Figure 4E, bottom panel). CTA mice consumed saccharin in significantly smaller bursts of licking than CTA control mice, as well as compared to that observed during water consumption (independent samples Mann-Whitney test, U = 1, *p* < 0.0001; dependent samples Wilcoxon test, CTA: *Z* =–2.80, *p* < 0.0001; Figure 4F, top). CTA control mice showed no significant differences in burst size compared to their performance during the water session (dependent samples Wilcoxon test, CTA control: *Z* =–0.66, *p* = 0.254; Figure 4F, bottom panel). The number of licks during the first 3 min of the session for the CTA group was significantly lower than that for the CTA control group on the retrieval day (independent samples Mann–Whitney U test, *U* = 22, p = 0.01; Figure 4G, top) and compared to the water day. The CTA control group showed no significant differences in licks between the water and retrieval days during the first 3 min (dependent samples Wilcoxon test: CTA: *Z* =–1.78, *p* = 0.037; CTA control: *Z* =–0.05, *p* = 0.48; Figure 4G, bottom). The frequency distribution of ILIs < 500 ms was fitted to a three-modal Gaussian curve for the CTA group and a bimodal Gaussian curve for the CTA control group (Figure 4H). CTA and CTA control mice displayed significant differences in the distribution of ILIs on saccharin retrieval day (two-sample KS test: *p* < 0.0001; Figure 4H). We examined several statistical parameters characterizing the distribution of ILIs during saccharin retrieval in both groups, as shown in the ILIs distribution histogram and indented pie chart (Figures 4H–J). The CTA group showed a significantly smaller proportion of ILIs within the first peak region (independent samples Mann–Whitney test: *U* = 2, *p* < 0.0001; Figure 4I) and an increased percentage of ILIs in the second peak region compared to the CTA control group (independent samples Mann–Whitney test: *U* = 1, *p* < 0.0001; Figure 4J). The distribution of ILIs also showed a third peak, with a significantly higher percentage of ILIs of > 300 ms in the CTA group than in the control group (Supplementary Figure 4H). The higher frequency of longer ILIs may imply hesitation in the CTA group. PCA was conducted, and a scatter plot was used to display the distribution of the CTA and control data (*n* = 20; Figure 4K). Bartlett’s sphericity test was used to determine the adequacy of the correlation matrix (Bartlett’s sphericity test: χ^2^ = 145.09, df = 36, *p* < 0.0001). Oblique factor rotation was performed after PCA, and the number of factors was chosen according to the explained proportion of the total variance (var. > 75%). The high communality (76%) of the data (Supplementary Figure 4J) (Budaev, 2010) allowed us to perform PCA on a relatively small dataset. Parallel analysis identified three principal components that collectively explained 76.13% of the variance between the groups (sample adequacy assessed using Bartlett’s test). PC1, PC2, and PC3 explained 34.83, 27.82, and 13.48% of the variance, respectively (Supplementary Figure 4L). The CTA and CTA control groups showed a reasonable separation in the PCA space, with 80% of the elements assigned to the right cluster (k-means algorithm). PC1 was negatively correlated with burst size and lick efficiency and positively correlated with burst count. PC2 was positively correlated with the lick count and initial licking rate (Figure 4K). These findings indicate that CTA mice exhibit an aversive pattern of licking behavior during saccharin retrieval, characterized by a reduction in total licks, licks during the first 3 min, burst size, increased burst count, and a percentage increase characterized by long ILIs, which reflects hesitant drinking.

### Licking microstructure analysis dissociates between innate and learned aversive tastants

Previous studies have reported that the total quinine intake is similar to that of learned aversive saccharin during CTA retrieval (Kayyal et al., 2019; Lavi et al., 2018). Given the advantage of licking microstructure analysis in providing more nuanced information on the parameters controlling taste valence, we hypothesized that microstructural analysis would reveal different patterns of drinking behavior associated with innate and learned aversive valences. Using the experimental design for CTA used in our previous studies, we compared quinine consumption (innate aversive group) and CTA retrieval (learned aversive group) during a 20-min licking recording session. The intake duration for quinine consumption was significantly longer than that for CTA retrieval (independent samples Mann–Whitney test: *U* = 0, *p* < 0.0001; Figure 5A). The total lick count was significantly higher in the learned aversion group than in the innate aversion group (Mann–Whitney U test: *U* = 15, *p* = 0.0068; Figure 5B). The burst count showed no significant difference between the learned and innate groups (independent samples Mann–Whitney test: *U* = 31, *p* = 0.0796; Figure 5C). The innate group showed significantly shorter licking bursts than the learned group (independent samples Mann–Whitney test: *U* = 6.5, p < 0.0001; Figure 5D). The number of licks during the first minute of consumption, a variable known to correlate with hedonic evaluation and CS palatability (Arthurs et al., 2012; Baird et al., 2005; Lin et al., 2013; Rebecca Glatt et al., 2016; Swick et al., 2015), was significantly lower in quinine than in CTA retrieval (independent samples Mann–Whitney test: *U* = 5, *p* < 0.0001; Figure 5E). The distributions of ILIs were fitted to a three-modal Gaussian curve for both the innate and learned groups. The two distributions were significantly different (two-sample KS test, *p* < 0.0001; Figure 5F). In addition, the percentage of ILIs within the first peak was significantly higher in the learned group than in the innate group (independent samples Mann–Whitney test: *U* = 2, *p* < 0.0001; Figure 5G). The proportion of ILIs > 180 ms was lower in the learned group than in the innate group (independent samples Mann–Whitney U test: *U* = 2, *p* < 0.0001; Figure 5H). To test the hypothesis that overall microstructure of licking behavior differs between innate and learned aversive tastes, in contrast to the simple measurement of consumption, we employed PCA. The dimensionality reduction of the principal component analysis for the innate and learned data is displayed using a scatter plot (*n* = 20; Figure 5I). Bartlett’s sphericity test was used to determine the adequacy of the correlation matrix (χ^2^ = 213.339, df = 36, *p* < 0.0001). The number of factors was chosen according to the proportion of the explained total variance (var. > 75%). The variables showed high communality (85%) (Supplementary Figure 5A), which allowed us to perform PCA using a relatively small sample size (Budaev, 2010). Following a parallel analysis, three principal components were selected, collectively explaining 83.95% of the variance among the valences. The learned and innate groups showed clear separation, with 90% of the elements assigned to the correct cluster using (k-means algorithm). PC1 explained 41.77% of the variance and was negatively correlated with lick efficiency and licks during the first 3 min. PC2 explained 27.13% of the variance and was negatively correlated with primary lick rate and intake duration. PC3 explained 15.05% of the variance, was negatively correlated with burst count, and positively correlated with pause duration (Supplementary Figures 5B,C). These results demonstrate quantifiable differences between two aversive tastes: innately aversive, as defined by evolution, and learned aversive, as defined by individual history.

## Discussion

Accurate assessment of an individual’s emotional reactions is challenging in both humans and animals. Taste elicits complex emotional responses that can be influenced by various factors, including palatability, incentive properties, post-ingestion processes, physiological states, and learning preferences or aversions (Naneix et al., 2020). Affective responses are important components of consumption. Preference tests for sweet tastants (e.g., saccharin or sucrose) are considered to reflect the animals’ capacity to experience hedonic pleasure evoked by sweet solutions (Berridge, 2004). However, for the proper measurement of taste emotional tagging, we need better tools than preference or avoidance assays.

Microstructural analysis of licking behavior has been used in various studies to assess different aspects of ingestive behavior. Recording the temporal organization of licks across consumption yields a rich dataset that can be analyzed to dissect consummatory responses into distinct licking patterns. The classical analysis approach involves examining parameters such as the number of bursts and burst size to measure palatability (Dwyer, 2012; Johnson, 2018). Despite providing valuable and significant insights into the factors regulating consumption, the conventional approach for analyzing the licking microstructure has several drawbacks. Different experimental conditions, variations in the choice of pause criterion, and the procedural and analytical methods used to classify bursts of licking have caused discrepancies in the literature (Johnson, 2018; Naneix et al., 2020). Other studies have used the orofacial taste reactivity test, which provides essential insights into the hedonic evaluation of taste stimuli but has limitations (Berridge, 2000; Naneix et al., 2020; Trnka et al., 2011). In this study, we sought to assess hedonic responses in freely behaving mice while drinking tastants in different contexts. We suggest a comprehensive analysis of fluid-licking patterns, including the temporal structure of bursts, ILI distribution histograms, and multivariate analysis. This multidimensional analysis revealed additional features of the licking pattern beyond those of classic microstructure analysis. We describe a simple, low-cost setup developed from commercially available components to record high-resolution, high-fidelity data and user-friendly custom-made software for collecting and analyzing data that we offer under an open-source license. Our software enables users to distill and export myriad behavioral parameters in various forms, allowing sophisticated microstructural analysis of the behavior in question with minimal computational effort. A pilot study with four independent experiments was conducted to test the reproducibility of a custom-built setup involving the licking behavior of 40 mice in response to water. The results showed comparable water consumption across all groups over time (Figure 2). Given that all temporal microstructural parameters of licking behavior are derived from the value chosen for the pause criterion, which varies among studies (Naneix et al., 2020), the only comparable measure we identified was the licking rate (licks/s). Our findings are consistent with those of previous studies (Johnson et al., 2010; Boughter et al., 2007; Boughter et al., 2012), thereby providing evidence of the validity of the system.

We sought to examine whether the affective valence of emotionally salient stimuli is innately determined and whether learning affects the innate value of the stimuli in freely behaving mice. We found that the mice responded to quinine (innate aversive), saccharin (innate appetitive), and water (neutral stimulus) with distinctive licking patterns. Second, although innate and learned aversive tastants elicited aversive behavior, microstructural analysis revealed a stronger aversion toward the innately aversive tastant than the learned one.

Currently, only a few studies conducted on rats have examined the licking microstructure of innate aversive tastes, specifically quinine (Hsiao and Fan, 1993; Spector and St John, 1998). In accordance with these studies, our findings showed that the aversive nature of quinine suppressed licking patterns compared with those of saccharin or water. Moreover, despite their high species diversity, rats and mice possess evolutionarily conserved abilities to experience emotions (Gu et al., 2018). The mice showed a dislike for quinine, as evidenced by their aversive licking pattern compared to that with water. Quinine led to a decrease in total licks, intake rate, and alterations in the distribution of ILIs, indicating scattering of licking responses and fragmentation of ingestive behavior. Interestingly, there were no differences in burst count and pause duration between quinine and water, which is consistent with a previous study in rats (Hsiao and Fan, 1993). However, this contradicts the findings of another study that reported increased bursts and decreased pause duration in rats (Spector and St John, 1998). These conflicting results could be attributed to variations in the study design, including differences in test duration and pause criteria. For example, employing a 500 ms pause criterion has been demonstrated to be effective in studies examining the licking microstructure in rats (Gutierrez et al., 2006), which contrasts with the 300 ms criterion supported by our data for the mice. This highlights the importance of customizing analytical parameters to specific species and experimental paradigms under investigation.

Studies using preference tests and those using the analysis of licking microstructures have assessed the hedonic evaluation of sweet solutions compared to neutral stimuli, such as water. Both methods yielded a wide range of results, from preference for sweet tastants to indifference, depending on factors such as solution concentration, prior animal experience, experimental conditions tailored to specific research interests, and differences in pause criteria (Johnson et al., 2010; Bachmanov et al., 2001; Grigson et al., 1993; Mendez et al., 2015; Monk et al., 2014; Rehn et al., 2023; Sclafani, 2006; Wong and Jones, 1981). Our findings showed no significant difference between water and saccharin in terms of total licks during the session, number of bursts, or number of licks made in the first minute (Figures 3B–D,F,G), but a significantly lower lick efficiency for saccharin than for water (Figure 3E).

Therefore, we expanded our analysis to examine additional parameters. Our study of ILIs distributions showed significant differences between water, saccharin, and quinine (Figures 3I–K), indicating that the ILIs from each test originated from a differentially distributed population. Our results replicated previous findings (Boughter et al., 2007; Johnson, 2018), with B6 mice displaying over 90% of licks occurring at interval of 60–180 ms, as they are thought to reflect continuous licking (Davis and Smith, 1992). This distribution is thought to be under the control of a central pattern generator (CPG) in the hindbrain and reflects rhythmic tongue movements in the rodent (Wiesenfeld et al., 1977). The ILIs distribution of saccharin showed a shift toward longer ILIs and produced a second distinguishable region of ILIs between 180 and 300 ms, and for quinine, ILIs > 300 ms produced a third region. A uniformly distributed shift to longer ILIs should have impacted the MPI in the same direction; however, our study shows that the MPI of the first range of saccharin is similar to that of water, and that the MPI of quinine decreases relative to both water and saccharin, suggesting that the longer ILIs within the primary distribution region (60–180 ms), possibly generated by hedonic evaluation processes (Johnson, 2018), are more likely to be shifted than the shorter primary ILIs produced by the CPG. This might explain why, although saccharin and quinine have opposite valence values, the ILIs histograms of both tastants show similar changes toward longer ILIs and shorter MPIs than water. Another plausible explanation is that the shift toward longer ILIs reflects the soft negative valence associated with the neophobic response induced by the first exposure to saccharin. Unbiased PCA and clustering demonstrated distinct microstructural patterns of licking behavior for innate aversive, appetitive, and neutral familiar taste stimuli. In summary, we found that mice responded to quinine (innate aversive) and saccharin (innate appetitive) with distinctive patterns of licking behavior.

As expected, we observed that innately aversive quinine elicited an aversive behavioral response. Similarly, because these responses can be acquired following experience, our study showed aversive behavior patterns toward aversively conditioned saccharin (Figure 3B–H), which is in agreement with (Lin et al., 2017; Rebecca Glatt et al., 2016). Studies that used outcome measures such as intake alone revealed that aversion indices for innately aversive quinine and learned aversive tastants (CTA) were indistinguishable (Kayyal et al., 2019; Lavi et al., 2018). Studies that have examined the microstructure of licking behavior in the CTA paradigm have reported that behavioral measures of hedonic evaluation were altered to resemble those typically associated with quinine (Arthurs et al., 2012; Baird et al., 2005; Dwyer, 2009, 2012; Dwyer et al., 2009; Lin et al., 2012, 2017; Lin et al., 2013; Swick et al., 2015). Studies have shown that different neural circuits and cellular mechanisms in the anterior insular cortex (aIC) modulate the retrieval of innate and learned aversive taste valences (Centanni et al., 2021; Schier and Spector, 2019; Schiff et al., 2018; Wu et al., 2020; Yiannakas et al., 2021). Accordingly, we aimed to determine whether and how microstructure analysis dissociates innate (quinine) and learned (CTA group) aversive tastes (i.e., different tastes with the same valence) in mice.

The initial lick rate and burst size reflect hedonic evaluations (Dwyer, 2012; Gero, 2020; St John et al., 2017). Using the same concentrations of tastant stimuli as in the CTA paradigm used in the aforementioned studies, we compared the licking patterns of innate and learned aversive stimuli and identified various reliable microstructural parameters that distinguish between innate and learned aversive taste valences. The innate aversive tastant (quinine) demonstrated a greater intensity of aversion than the learned tastant (CS). One possible explanation for this is that innately aversive tastants are more resistant to changes in their motivational domains (Schier and Spector, 2019). Another plausible explanation for these results is that innate and learned valences are encoded by distinct signaling pathways and brain regions (Eschbach et al., 2021; Inui et al., 2007; Ramírez-Lugo et al., 2016).

Our findings indicate that a multidimensional analysis provides a more thorough method for examining licking behavior. This methodology reliably distinguishes subtle differences in taste valence, thereby expanding the behavioral repertoire that can be measured in freely moving mice to include taste. A limitation of the present study is the exclusive use of male animals. This methodological choice was made to minimize the potential behavioral variability associated with hormonal fluctuations of the estrous cycle in females, which are known to influence ingestive behavior (Eckel, 2011). However, we acknowledge that this limitation affects the generalizability of our findings. There is increasing evidence of sex-specific differences in both innate taste preferences and learned taste aversion (Becker and Koob, 2016). For instance, some studies have reported that female rodents may acquire or express conditioned taste aversions differently than males do (Choleris et al., 2000). Therefore, the distinctive microstructural licking patterns we identified may be specific to males. An important direction for future research will be to incorporate both sexes to determine the broader applicability of these findings to both sexes. Future studies could combine measurements of the dynamics of taste sensory information and licking microstructures with measurements of neuronal activity in relevant brain structures (e.g., Lavi et al., 2018) in real time to gain a better understanding of the underlying mechanisms that enable the encoding of the plasticity of taste emotional tagging.

## Data Availability

The original contributions presented in the study are included in the article/Supplementary material, further inquiries can be directed to the corresponding authors.

## Footnotes

1
https://www.stat.ubc.ca/~rollin/stats/ssize/n2.html

2
https://github.com/randasal/Taste-Software-2025
