# Supplementary material for: Licking microstructure behavior classifies a spectrum of emotional states in mice
Source: Front Syst Neurosci. 2025 Aug 13;19:1623084. doi: 10.3389/fnsys.2025.1623084 (PMC12380781; doi:10.3389/fnsys.2025.1623084)
Supplement: Supplementary file 1 [file Data_Sheet_1.pdf]

Supplementary Material

1 Supplementary Data

Supplementary File 1.

Software User Guide

Disclaimer of Warranty

This software is provided "as is", without warranty of any kind, express or implied, including but not limited to the warranties of merchantability, fitness for a particular purpose and non-infringement. In no event shall the authors or copyright holders be liable for any claim, damages or other liability, whether in an action of contract, tort or otherwise, arising from, out of or in connection with the software or the use or other dealings in the software.

This clause is in accordance with the MIT License, under which this software is released.

Introduction

The Taste System Software is implemented in Matlab and consists of 2 distinct programs (each comprises several modules so the total number of code files is larger)

- ***TasteExtractData*** – Extracts experiment data from the recording system (***Intan, Davis or TDT photometry system***) files and converts them into Matlab files, ready for data analysis.

- ***TasteAnalysisSW*** - Data Analysis software designed to generate all sorts of activity presentation graphs, data characterization and statistical analysis reports. In addition, the system can export the data into excel files organized in a way that will make it easy to be imported into professional statistical packages.

In addition to the home-grown modules, the ***TasteExtractData software*** is using Matlab files provided by Intan and TDT to convert their proprietary binary files to Matlab structures which (during the extraction process) are repackaged in our proprietary format, and stored as Matlab data files.

Installation

The Taste System SW can be downloaded from GitHub. The Github repository is defined as a private one. In order to download, therefore, a Github account is required and an access permission from randa.sa@gmail.com

It is organized in a single directory TatstSystemSW which can be placed anywhere in the user space (see example in **Error! Reference source not found.**). No special installation and/or registration are required.

2 Extract Data

The Extract Data module extracts behavioral and neuronal data from the recording system files, then converts into Matlab data (mat) files which are stored in the relevant directories. This program is using conversion libraries provided by Intan (embedded in our code) and TDT (needs to be copied into the SW directory tree, see Installation section above). Data is extracted from all recorded data present in the recorded data directory. A single Matlab file is generated, for each extraction. A screen shot of the program GUI is provided in Figure 7.

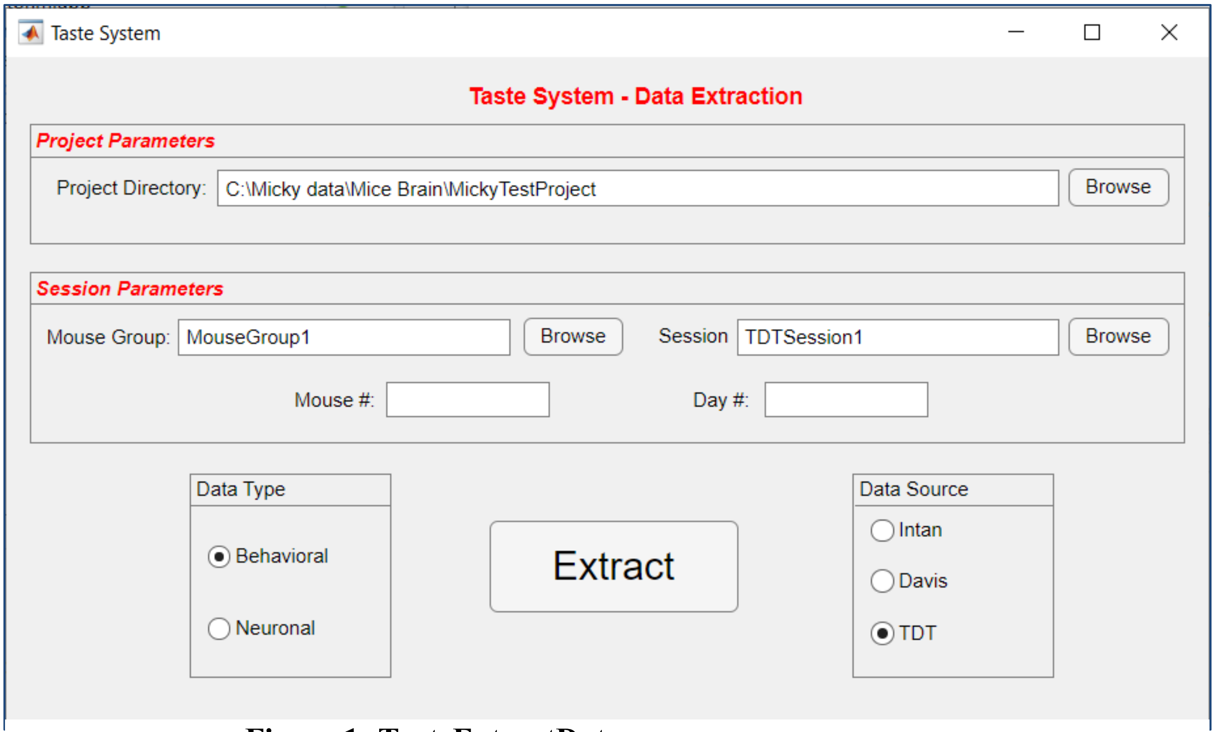

Figure 1: TasteExtractData

3 Taste Analysis

The TasteAnalysis module produces a set of reports out of recorded data. Depending on the requested output, the input may include behavioral and neuronal data from single or multiple mice. The following sections describe the user interface of the module and the reports it is designed to generate. A screen shot of the program GUI is provided in

Figure 2.

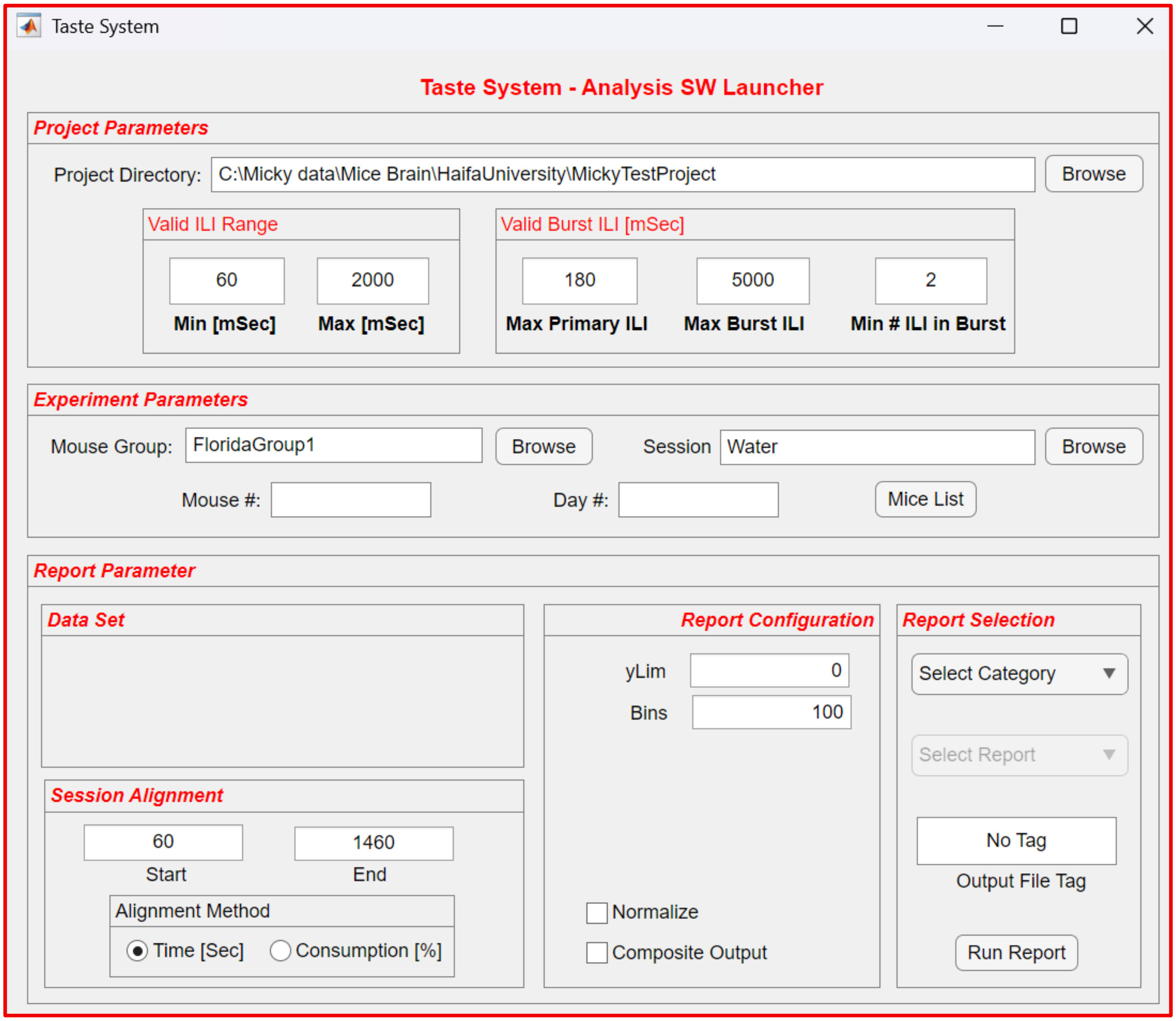

Figure 2 - Taste Analysis GUI

4 Supplementary Figures and Tables

4.1 Supplementary Figures

A

Communalities		
**Variables**	**Initial**	**Extraction**
**Lick Count**	1.000	4.564
**Burst Count**	1.000	2.724
**Burst Size**	1.000	0.945
**Primary Rate**	1.000	0.442
**Effective Rate**	1.000	0.197
**Lick Efficiency**	1.000	0.072
**Average (ILI)**	1.000	0.052
**STD (ILI)**	1.000	0.003
**Mean Primary ILI (MPI)**	1.000	0.001

B

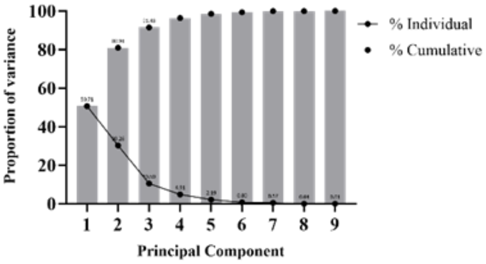

C

Principal component analysis (PCA) loadings			
**Variables**	**PC1**	**PC2**	**PC3**
**Lick Count**	**0.798293**	-0.43761	0.356879
**Burst Count**	-0.16292	**-0.70456**	**0.600198**
**Burst Size**	**0.911373**	-0.11192	-0.18394
**Primary Rate**	**-0.67066**	**0.617244**	0.327404
**Effective Rate**	0.034092	**0.919521**	0.367146
**Lick Efficiency**	**0.792211**	0.53245	0.113273
**Average (ILI)**	**-0.69612**	**-0.63962**	-0.1211
**STD (ILI)**	**-0.8462**	-0.1339	0.276911
**Mean Primary ILI (MPI)**	**0.88904**	-0.2938	0.278809

Supplementary Figure 3.
(A) Table of communalities of PCA. (B) Table of PCA loadings. (C) PCA proportion of variance.

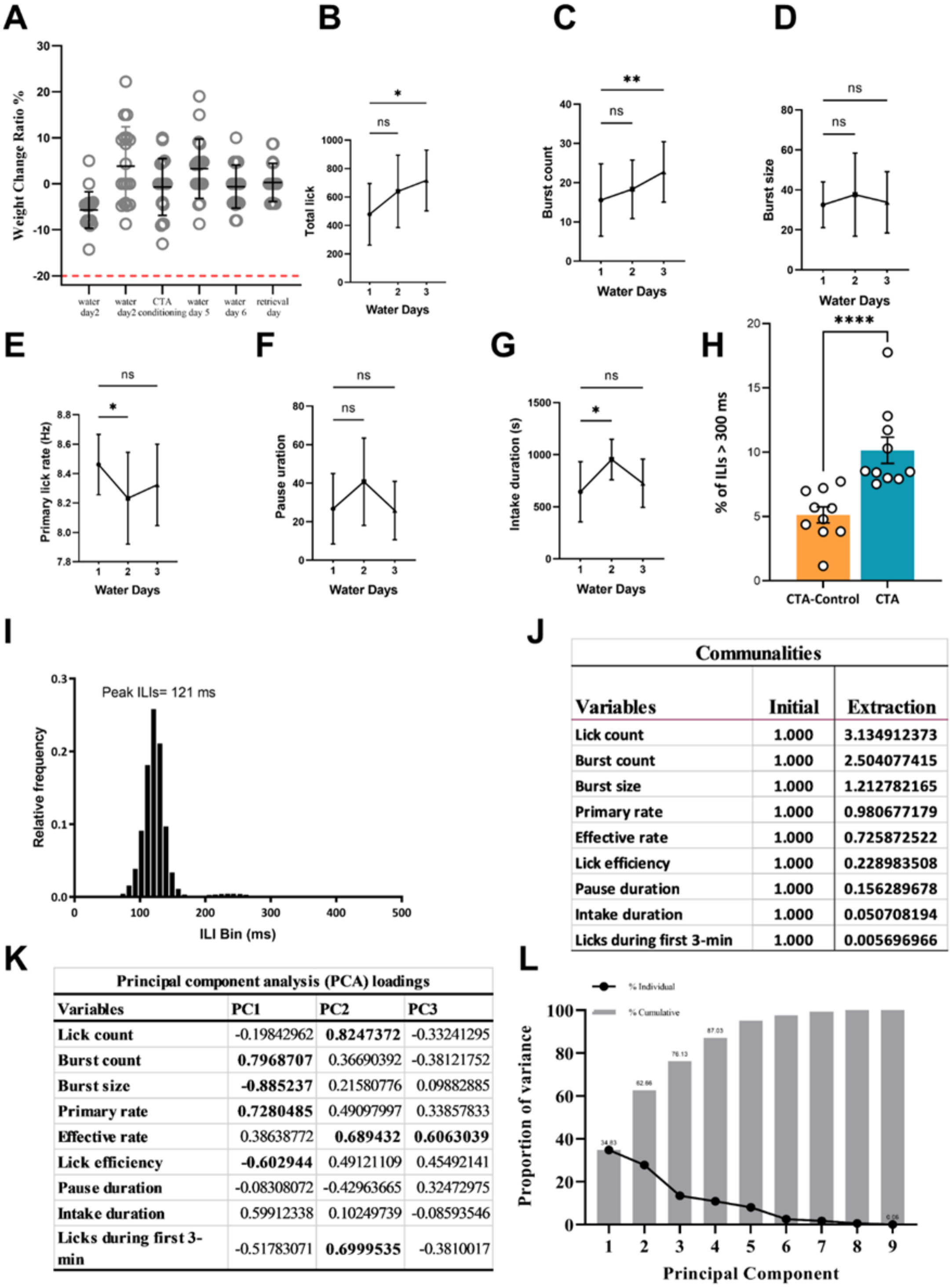

Communalities		
**Variables**	**Initial**	**Extraction**
Lick count	1.000	3.134912373
Burst count	1.000	2.504077415
Burst size	1.000	1.212782165
Primary rate	1.000	0.980677179
Effective rate	1.000	0.725872522
Lick efficiency	1.000	0.228983508
Pause duration	1.000	0.156289678
Intake duration	1.000	0.050708194
Licks during first 3-min	1.000	0.005696966

Principal component analysis (PCA) loadings			
**Variables**	**PC1**	**PC2**	**PC3**
Lick count	-0.19842962	**0.8247372**	-0.33241295
Burst count	**0.7968707**	0.36690392	-0.38121752
Burst size	**-0.885237**	0.21580776	0.09882885
Primary rate	**0.7280485**	0.49097997	0.33857833
Effective rate	0.38638772	**0.689432**	**0.6063039**
Lick efficiency	**-0.602944**	0.49121109	0.45492141
Pause duration	-0.08308072	-0.42963665	0.32472975
Intake duration	0.59912338	0.10249739	-0.08593546
Licks during first 3-min	-0.51783071	**0.6999535**	-0.3810017

Supplementary Figure 4.

(A) Weight Change Ratio graph for the two groups. Each gray dot represents a single mouse weight variation compared with the previous day. The black line indicates the means and S.E.M; the values are specified above the graph for each day. The red dashed line represents the decrease of 20% of body weight, the limit below which the animal is in severe suffering (WD 2: -5.68 ± 0.90; WD 3:3.84 ± 1.96; CTA day: -0.70 ± 1.38; WD 5:3.27 ± 1.44; WD 6: -0.62 ± 1.04; RD:0.28 ± 0.92). (B) Total lick (WD

1: 478.7 ± 65.32; WD 2: 640 ± 76.85; WD 3: 717 ± 64.43). (C) Burst count (WD 1: 15.58 ± 2.65; WD 2: 18.33 ± 2.15; WD 3: 22.75 ± 2.22). (D) Burst size (WD 1: 32.59 ± 3.31; WD 2: 37.66 ± 6; WD 3: 33.85 ± 4.43). (E) Primary lick rate (WD 1: 8.46 ± 0.06; WD 2: 8.23 ± 0.09; WD 3: 8.32 ± 0.08). (F) Pause duration (WD 1: 26.7 ± 5.27; WD 2: 4.74 ± 6.55; WD 3: 25.81 ± 4.38). (G) Intake duration (WD 1: 644.1 ± 83.33; WD 2: 954.3 ± 56.3; WD 3: 725.9 ± 66.95). (H) Comparison of the mean percentage of ILIs > 300 ms between the CTA (9.89%) and CTA control (4.38%) groups. (I) ILIs bin distribution for water training days. The frequency distribution of ILIs < 500 ms (10 ms bins) showed a distribution with a mode of 121 ms; the second peak was approximately double the modal value of the primary distribution. (J) Table of Communality of PCA. (K) Table of PCA loadings. (L) PCA proportion of variance

A

Communalities

Variables	Initial	Extraction
Lick count	1.000	3.757715826
Burst count	1.000	2.442981158
Burst size	1.000	1.354407764
Primary rate	1.000	0.845456924
Effective rate	1.000	0.291688794
Lick efficiency	1.000	0.219313653
Pause duration	1.000	0.080527772
Intake duration	1.000	0.005078388
Licks during first 3-min	1.000	0.002829721

B

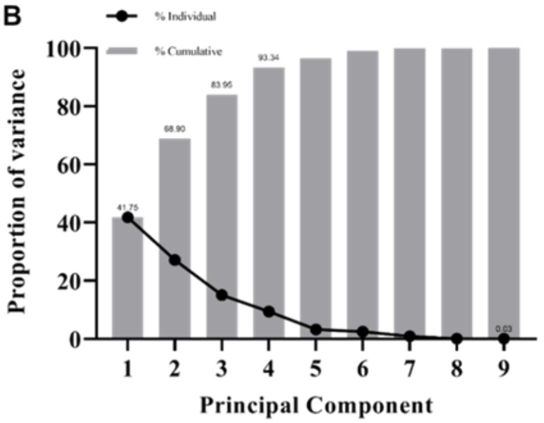

C

Principal component analysis (PCA) loadings

Variables	PC1	PC2	PC3
Lick count	**-0.935369**	0.0609748	-0.180219
Burst count	**-0.810227**	-0.280528	-0.426237
Burst size	-0.578137	0.5610318	0.3744172
Primary rate	-0.186128	**-0.920101**	-0.112358
Effective rate	**-0.604343**	**-0.666131**	0.2733834
Lick efficiency	**-0.69265**	-0.053701	0.5267893
Pause duration	0.2661074	-0.303866	**0.7958679**
Intake duration	0.279958	**-0.795641**	-0.040489
Licks during first 3-min	-0.929119	0.1650179	-0.012138

Supplementary Figure 5.
(A) Table of communalities of PCA (B) Table of PCA loadings (C) PCA proportion of variance

4.2 Supplementary Tables

Supplementary Table 1. Bill of Materials

Category	Component	Description & Function	Manufacturer (Product #)	Qty	Approx. Cost (USD)	Source (URL / Supplier)
**I. Behavioral Apparatus**	Acrylic Cage	Custom-fabricated transparent testing chamber (30×14.5×16 cm) for the subject.	Custom-Built (Lab Workshop)	1	20	N/A
	Wooden Cabinet	Sound-attenuating and electrically shielded (Faraday cage) enclosure for the behavioral apparatus.	Custom-Built	1	~600	N/A
	Aluminum Foil	Conductive ground plane lining the cage floor, essential for the lick-detection circuit.	DigiKey	1	20	Generic/Hardware Store
	Sipper Bottle	Glass drinking vial (25 ml) with a stainless-steel spout for delivering liquid solutions.	Med Associates (MED-DAV-250BT-M)	10	70	https://med-associates.com/product/sipper-bottle/
	IR Camera	Infrared camera for continuous monitoring of mouse behavior and well-being during experiments.	Generic	1	200	
**II. Lick Detection Hardware**	Contact Lickometer Controller	Single-channel controller that detects tongue-to-spout contact via circuit completion and generates a 28V DC output signal.	Med Associates (MED-ENV-250)	1	192	https://med-associates.com/product/contact-lickometer-controller/
	28V DC to TTL Adapter	Converts the 28V DC output from the lickometer controller into a 5V TTL signal suitable for digital data acquisition.	Med Associates (MED-SG-231)	1	222	https://med-associates.com/product/ttl-28v-dc-adapter/
	DC Power Supply	Provides stable 28V, 1A DC power to the lickometer controllers and associated modules.	Med Associates (MED-SG-501A)	1	346	
**III. Data Acquisition System**	Intan RHD2000 System	A multi-channel data acquisition system used to record the digital 5V TTL pulses (lick events) with high temporal resolution.	Intan Technologies (RHD 2000)	1	~1500	https://intantech.com/
**Total Estimated Cost**					~3170	
